# Optimization of 3D printing and *in vitro* characterization of alginate/gelatin lattice and angular scaffolds for potential cardiac tissue engineering

**DOI:** 10.3389/fbioe.2023.1161804

**Published:** 2023-05-25

**Authors:** Farinaz Ketabat, Titouan Maris, Xiaoman Duan, Zahra Yazdanpanah, Michael E. Kelly, Ildiko Badea, Xiongbiao Chen

**Affiliations:** ^1^ Division of Biomedical Engineering, University of Saskatchewan, Saskatoon, SK, Canada; ^2^ Institut Catholique des arts et métiers (ICAM)- Site de Toulouse, Toulouse, France; ^3^ Department of Surgery, College of Medicine, University of Saskatchewan, Saskatoon, SK, Canada; ^4^ College of Pharmacy and Nutrition, University of Saskatchewan, Saskatoon, SK, Canada; ^5^ Department of Mechanical Engineering, University of Saskatchewan, Saskatoon, SK, Canada

**Keywords:** three-dimensional (3D) printing, cardiac tissue engineering, printability, alginate, gelatin, fiber orientation

## Abstract

**Background:** Engineering cardiac tissue that mimics the hierarchical structure of cardiac tissue remains challenging, raising the need for developing novel methods capable of creating structures with high complexity. Three-dimensional (3D)-printing techniques are among promising methods for engineering complex tissue constructs with high precision. By means of 3D printing, this study aims to develop cardiac constructs with a novel angular structure mimicking cardiac architecture from alginate (Alg) and gelatin (Gel) composite. The 3D-printing conditions were optimized and the structures were characterized *in vitro*, with human umbilical vein endothelial cells (HUVECs) and cardiomyocytes (H9c2 cells), for potential cardiac tissue engineering.

**Methods:** We synthesized the composites of Alg and Gel with varying concentrations and examined their cytotoxicity with both H9c2 cells and HUVECs, as well as their printability for creating 3D structures of varying fibre orientations (angular design). The 3D-printed structures were characterized in terms of morphology by both scanning electron microscopy (SEM) and synchrotron radiation propagation-based imaging computed tomography (SR-PBI-CT), and elastic modulus, swelling percentage, and mass loss percentage as well. The cell viability studies were conducted via measuring the metabolic activity of the live cells with MTT assay and visualizing the cells with live/dead assay kit.

**Results:** Among the examined composite groups of Alg and Gel, two combinations with ratios of 2 to 1 and 3 to 1 (termed as Alg2Gel1 and Alg3Gel1) showed the highest cell survival; they accordingly were used to fabricate two different structures: a novel angular and a conventional lattice structure. Scaffolds made of Alg3Gel1 showed higher elastic modulus, lower swelling percentage, less mass loss, and higher cell survival compared to that of Alg2Gel1. Although the viability of H9c2 cells and HUVECs on all scaffolds composed of Alg3Gel1 was above 99%, the group of the constructs with the angular design maintained significantly more viable cells compared to other investigated groups.

**Conclusion:** The group of angular 3D-ptinted constructs has illustrated promising properties for cardiac tissue engineering by providing high cell viability for both endothelial and cardiac cells, high mechanical strength as well as appropriate swelling, and degradation properties during 21 days of incubation.

**Statement of Significance:** 3D-printing is an emerging method to create complex constructs with high precision in a large scale. In this study, we have demonstrated that 3D-printing can be used to create compatible constructs from the composite of Alg and Gel with endothelial cells and cardiac cells. Also, we have demonstrated that these constructs are able to enhance the viability of cardiac and endothelial cells via creating a 3D structure mimicking the alignment and orientation of the fibers in the native heart.

## GRAPHICAL ABSTRACT

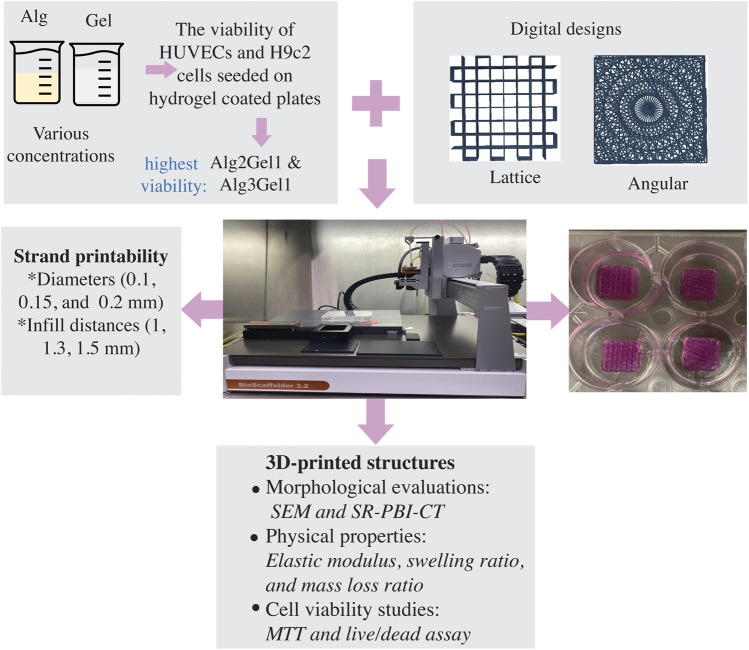

## 1 Introduction

Cardiovascular diseases (CVDs) are the leading cause of death, and their treatments remain a challenge. Due to the limited regenerative capability of myocardium and the lack of treatments to regenerate damaged myocardium, tissue engineering of such a complex tissue seems vital (Ozbolat, 2016; Hashimoto et al., 2018; Hendrickson et al., 2021). Cardiac tissue engineering (CTE) aims to fabricate heart-mimicking constructs by engineering methods, thus offering a promising tool for the secondary management of CVDs, especially after myocardial infarction and congestive heart failure (He and Chen, 2020; Pomeroy et al., 2020). Despite progress in the field of CTE, fabricating constructs with the heart hierarchical properties (i.e., ranging from detailed molecular signaling pathways to cardiac function) of the native myocardium and the branched vascular network within the tissue is still challenging (Gould et al., 2013; Roy et al., 2018).

Despite all the significant advances in the field of CTE, there are still many challenges that need to be addressed. The primary challenges in CTE are 1) vasculogenesis -for efficient exchange of oxygen between the cells and blood- and 2) mimicking the complex structure of the myocardium (Vunjak-Novakovic et al., 2011; Izadifar et al., 2014; Kitsara et al., 2017). The orientation of the extracellular matrix fibrils can change vascularization by affecting the migration and polarization of endothelial cells, their branching, formation of basement membrane, and cellular traction (Labelle et al., 2022). Thus, to replicate the complex structure of the human heart tissue, many parameters including alignment of the cells within the fibrils should be appropriately designed and controlled (Zhang et al., 2015; Qasim et al., 2019). The geometry of the scaffold substrate plays a vital role in CTE since cardiac tissues must be highly differentiated to function properly (Nguyen et al., 2019). Detailed histological maps of the human heart revealed that the orientation of the fibres in the outer wall of the heart is −70° and it gradually increases to + 80° in the inner wall (Wong and Kuhl, 2014).

Three-dimensional (3D) printing is a promising technique that may revolutionize tissue engineering and regeneration. This method provides an opportunity for the accurate deposition of materials loaded with different cells in a layer-by-layer approach to create complex tissue-engineered constructs, mimicking the complexity of the native tissue (Mosadegh et al., 2015; Roopavath and Kalaskar, 2017; Chen, 2019; Carvalho et al., 2021; Yazdanpanah et al., 2022). Nowadays, 3D printing has shown to be a promising approach for fabrication of functional cardiac constructs with appropriate structures and properties (Izadifar et al., 2017a; Agarwal et al., 2021; Delkash et al., 2021).

Hydrogels have attracted considerable attention in 3D-printing for CTE. Hydrogels can induce vascularization through micropatterning and can direct the alignments of cardiac cells to improve the function of the cardiac construct (Camci-Unal et al., 2014). Among various biomaterials, alginate is a promising candidate to make hydrogels for cardiac tissue engineering due to its physical and mechanical properties comparable to the native cardiac tissue (Izadifar et al., 2017a; Mirdamadi et al., 2020). While the elastic modulus of heart tissue ranges from 10 kPa to 50 kPa, the elastic modulus of alginate scaffolds can be tuned ranging from 10 kPa to 40 kPa, by altering its molecular weight, crosslinking with calcium ions, and partial oxidation (Izadifar et al., 2017a; Sarker et al., 2018; Mirdamadi et al., 2020; Hendrickson et al., 2021). Alginate is a biocompatible, nonimmunogenic hydrogel with extracellular matrix-like characteristics that make this material a suitable candidate for tissue repair and regeneration (Kumar et al., 2021). Besides its low cost, alginate has the merit to be 3D printed into complex and small structures with high fidelity (Hinton et al., 2015; Izadifar et al., 2017a; Mirdamadi et al., 2020). However, alginate is not a native component of the heart that does not support fibronectin adhesion (McCain et al., 2014). Gelatin is a derivative of collagen-the main component of the extracellular matrix of many organs, such as the heart-which can support cell adhesion (McCain et al., 2014). Unlike collagen, gelatin does not express antigenicity in physiological conditions, and it is an inexpensive hydrogel compared to collagen (Rosellini et al., 2009). However, gelatin lacks stability in physiological conditions and has poor mechanical properties (Xing et al., 2014). The synergistic properties of alginate/gelatin composites on cell viability and printability make them attractive inks for 3D printing and tissue engineering (Soltan et al., 2019; You et al., 2020).

Considering the advantages of alginate and gelatin for tissue engineering applications and their low cost, these hydrogels are significantly unexplored. There are only a few studies focusing on developing cardiac tissues using alginate and gelatin. It was found that alginate and gelatin can be used as a bioink to 3D-bioprint cardiac spheroids into lattice constructs and grow cardiac tissue following 7–13 days after the printing (Zhou et al., 2016). Later, it was found that the constructs could significantly improve cardiac function in the mice modeling myocardial infarction (MI). Interestingly, even acellular 3D constructs alone partially enhanced heart function (Maiullari et al., 2018a). Another study reported that alginate/gelatin could enable the fusion of cardiac spheroids, showing their potential to be used as a long-term *in vitro* heart model (Dominijanni et al., 2021). Although these studies were successful at growing cardiac spheroids using 3D-printing techniques, they used a conventional lattice design (0, 90°) for their constructs. Therefore, studies focusing on design of constructs that mimic the internal angular structure of the myocardium are lacking. Although one of the studies investigated the viscoelasticity of their scaffolds (Dominijanni et al., 2021) and the other studied the durability of their constructs (Zhou et al., 2016), detailed physical and mechanical behaviour of their scaffolds were not evaluated. Therefore, developing cardiac constructs that mimic the internal structure of myocardium to grow a more myocardium-like structure following their *in vitro* optimization and characterization is necessary.

The present study aims to 3D-print alginate/gelatin constructs that mimic the orientation of fibers in the native myocardium and characterize them *in vitro* for potential cardiac tissue engineering. The hypothesis is that the design will result in structures with mechanical strength and configuration more similar to the ventricle compared to the lattice design. Therefore, these constructs could provide a better support for ventricle tissue once they are transplanted *in vivo*. Due to the similarity of this design with myofibrils, it could further be used for 3D-bio printing of cell-laden alginate/gelatin hydrogels to grow a 3D model for cardiac tissue *in vitro.* H9c2 cells are used as an *in vitro* cell model for cardiomyocytes due to their contractile properties which make them a suitable model where large qualities of cells are needed (Zhou et al., 2016) To engineer a cardiac tissue model enriched with a vascular network (Maiullari et al., 2018a), human umbilical vein endothelial cells (HUVECs) are employed. HUVECs can form blood vessel-like tubules inside the engineered cardiac tissue that can be integrated with host’s vascular network (Maiullari et al., 2018a). Specifically, this study focuses on 1) characterization of the biological and printability properties of alginate/gelatin composites to find the optimum parameters for fabricating 3D cardiac constructs; 2) design of a cardiac construct that mimics the orientation of the fibres in the native human heart and tuning the 3D-printing parameters to fabricate this construct; and 3) characterization of the 3D constructs in terms of physical and morphological properties and viability of the seeded cardiac and endothelial cells onto the 3D-printed constructs.

## 2 Material and experimental methods

### 2.1 Hydrogel preparation

Composite hydrogels were prepared from alginate and gelatin in six (6) ratios. Gelatin at three different concentrations of 1, 2, and 3 wt% (Type A, gel strength ∼300 g Bloom, Sigma-Aldrich) was dissolved in saline solution (0.9 %w/v sodium chloride in Milli-Q water) at ∼ 55°C, while solutions of 2 and 3 wt% alginic acid sodium salt were prepared from brown algae (medium viscosity, Sigma-Aldrich). The obtained solutions of alginate and gelatin were then mixed to form composite hydrogels with 6 ratios, which were alginate 2 wt%/gelatin 1 wt% (Alg2Gel1), alginate 2 wt%/gelatin 2 wt% (Alg2Gel2), alginate 2 wt%/gelatin 3 wt% (Alg2Gel3), alginate 3 wt%/gelatin 1 wt% (Alg3Gel1), alginate 3 wt%/gelatin 2 wt% (Alg3Gel2), and alginate 3 wt%/gelatin 3 wt% (Alg3Gel3). Also, solutions of alginate or gelation alone, i.e., alginate 2 wt% (Alg2), alginate 3 wt% (Alg3), gelatin 1 wt% (Gel 1), gelatin 2 wt% (Gel 2), and gelatin (Gel 3) were prepared for the subsequent experiments.

### 2.2 Cell culture

HUVECs (ATCC 1730-CRL) were maintained in complete culture media including, DMEM (SH30243.01, HyClone, Cytiva) supplemented with 10% fetal bovine serum (FBS, Gibco^®^, Invitrogen) and 1% antibiotic-antimycotic (100X) containing 10,000 units/mL penicillin, 10,000 μg/mL of streptomycin, and 25 μg/mL of Fungizone (Thermo Fisher) and 2% HAT (50X) (sodium hypoxanthine, aminopterin and thymidine). H9c2 cells (ATCC CRL-1446) were maintained in DMEM supplemented with 10% fetal bovine serum and 1% antibiotic-antimycotic (100X), and 2% HAT (50X). The cells were grown in a humidified incubator at 37°C with 5% CO_2_. Cells detachments were done with a solution of 0.25% trypsin-EDTA (Gibco^®^, Invitrogen).

### 2.3 Cell viability assays for hydrogels

The bottom of 96-well plates was coated with 50 µL of each hydrogel solution. HUVECs were seeded on hydrogel samples (0.8 × 10^4^ cells per well). After 48 h of seeding, the viability of cells was studied by MTT (3-(4,5-Dimethylthiazol-2-yl)-2,5-Diphenyltetrazolium Bromide, M6494, Invitrogen, ThermoFisher). The assay was repeated three times, each performed in five replicates (n = 15). MTT in fresh media (0.1 mg/mL) was added to each well. After 2 hours of incubation at 37°C, dimethyl sulfoxide (DMSO) was added to each sample. Then, the plates were placed on a shaker for 2 hours and the absorbance was read at wavelength of 550 nm using a BioTek Synergy HT Multi-Detection Microplate Reader. The same procedure was repeated for H9c2 cells as well. Cells seeded on the plate without any hydrogel were considered as control.

The viability of the cells was also studied using Live/Dead assay as a confirmatory approach. Due to the nature of 3D cell culture, the quantitative data have the potential to show errors (Dominijanni et al., 2021). Notably, the combination of quantitative data with microscopic imaging is suggested for complementary examination of 3D cell culture (Dominijanni et al., 2021). For this, in the present study, the cells were stained with calcein AM (AnaSpec, Fremont) and propidium iodide (PI, AnaSpec, Fremont) after 48 h of seeding. A solution containing DMEM, calcein Am (1 μg/mL: to stain live cells) and PI (10 μg/mL: to stain dead cells) was added to each sample and incubated for at least 40 min (Ning et al., 2016) at room temperature. Images were taken using ZOE Fluorescent Cell Imager (BIO-RAD).

### 2.4 3D-printing the hydrogels

**FIGURE 1:**
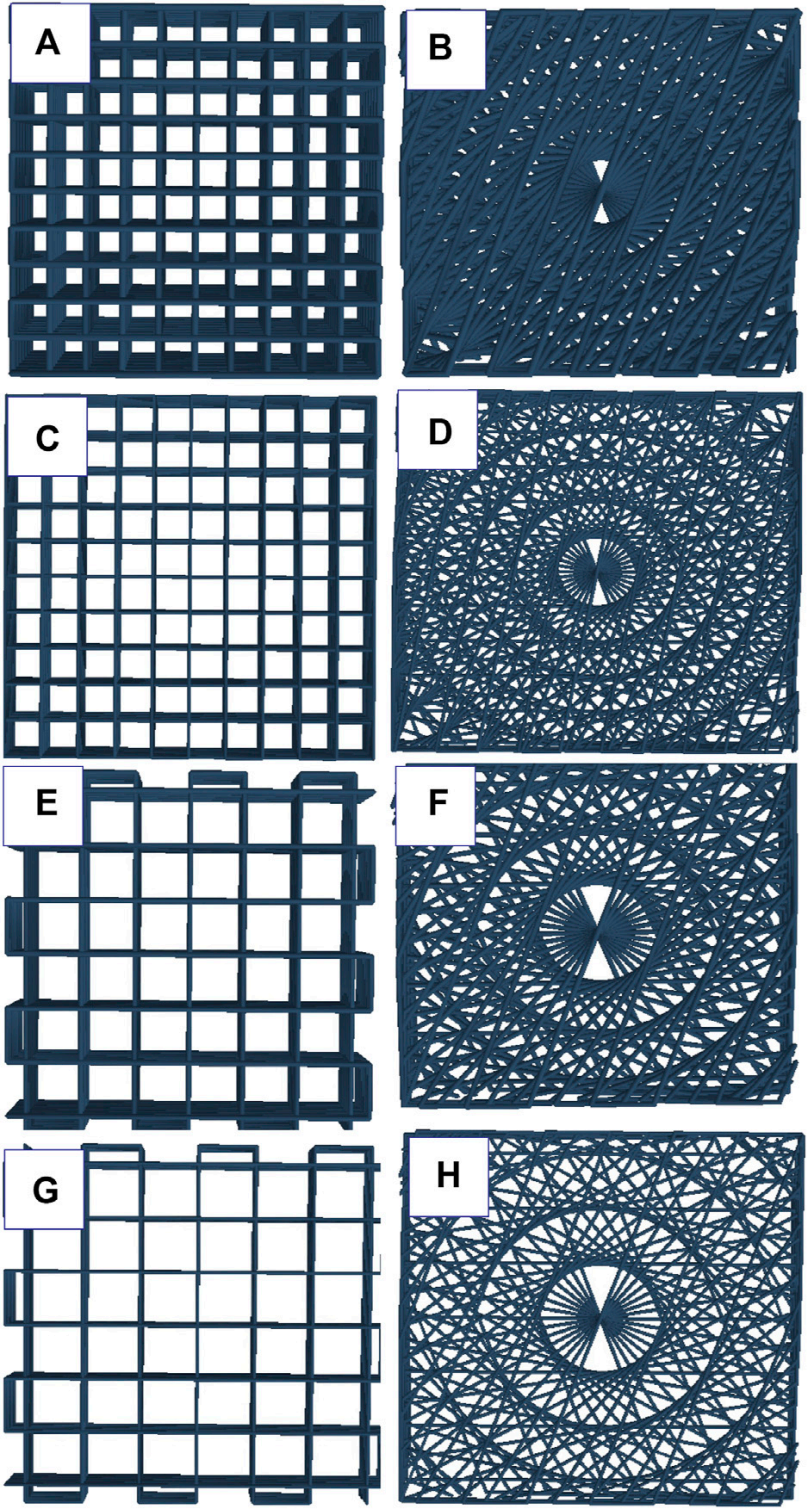
schematic representation of angular and lattice structures: **(A)** Alg2Gel1[1,0.2]-L, **(B)** Alg2Gel1[1,0.2]-A, **(C)** Alg2Gel1[1,0.1]-L, **(D)** Alg2Gel1[1,0.1]-A, **(E)** Alg3Gel1[1.5,0.15]-L, **(F)** Alg3Gel1[1.5,0.15]-A, **(G)** Alg3Gel1[1.5,0.1]-L, **(H)** Alg3Gel1[1.5,0.1]-A.

A box-shaped structure (10 mm × 10 mm) was created using 3D builder software in a 3D manufacturing format (3 MF). The slice thickness was defined as 80% of the theoretical strand diameter. Gesim Robotics (Version 1.17.1.4352 ^©^2016Gesim Robotics) was used to slice the model into a 15- printable layer structure. Two types of scaffolds were printed; I) Angular structure: the first layer deposited at a −70° angle with a +10° change in each layer. II) Lattice structure: the samples were printed in a perpendicular pattern with alternating angles of 0° and 90° in each layer (Figure 1). The scaffolds were printed using a BioScaffolder 3.2 (Gesim, Germany). The samples were printed into 12-well plates. Due to the presence of carboxyl groups with negative charges in native alginate (Lin et al., 2019), the plates were coated with 0.1% polyethyleneimine (PEI, M.W. 60,000, 50% w/w aq. Thermo Scientific) at 37° the day before the 3D printing. PEI is a highly positively charged polycation which can easily react with alginate and stabilize its molecular structure by forming a polyelectrolyte complex (Rajaram et al., 2015; Chen et al., 2020). This treatment leads to the enhanced attachment of alginate strands to the surface of the plate (Rajaram et al., 2015; Naghieh et al., 2018). The next day, the PEI solution inside the wells was replaced with 50 mM calcium chloride (CaCl_2_) in 0.1% PEI solution and the scaffolds were 3D-printed inside the solution. All the scaffolds (except for the printability evaluation study) were 3D-printed using a 27G SmoothFlow Tapered Tip with an inner diameter of 0.2 mm (EFD, Nordson) into 12 well plates. Then they were incubated with 2 mL of 100 mM CaCl_2_ solution for 10 min immediately after 3D-printing, washed three times with 1X phosphate-buffered saline (PBS), and maintained in complete culture media.

### 2.5 Printability evaluation

Printability, referred to the ability to form reproducible 3D scaffolds from the same ink or bioink, is a critical performance index in 3D-extrusion-based printing (Naghieh and Chen, 2021). Scaffold design, bioink properties, and printing processes such as speed, pressure, and crosslinking mechanism can affect printability (Fu et al., 2021; Naghieh and Chen, 2021). Examining the difference between the strand diameter in a 3D-printed structure *versus* the designed one is an example of widespread practice to measure printability (Fu et al., 2021).

To design reproducible 3D scaffolds, nine distinctive designs with different infill distances (the distance from the middle of one strand to the adjacent strand) and strand diameters were 3D-printed for both Alg2Gel1 and Alg3Gel1 with angular and lattice structures. The samples are coded as Alg2Gel1[a,b]-L, Alg2Gel1[a,b]-A, Alg3Gel1[a,b]-L, Alg3Gel1[a,b]-A where a, b, A, L represent infill distance, strand diameter, angular structure, and lattice structure, respectively. Considering three infill distances (1.0, 1.3, 1.5 mm) and three strand diameters (0.1, 0.15, 0.2), 36 different designs were 3D-printed in triplicates. Two layers of strands were 3D-printed under a constnt printing speed (6 mm/s for Alg2Gel1 and 4 mm/s for Alg3Gel1) and pressure (10 kPa for Alg2Gel1 and 20 kPa for Alg3Gel1).

Three images from different areas of the scaffolds were taken using Leica light microscope immediately after 3D-printing. The diameter of each strand was measured using ImageJ^®^ software (National Institute of Health, Bethesda, MD, USA). The strand printability was calculated by Eq. 1 (Naghieh et al., 2019).

$$Strand\;printability=1-\left(\frac{{d_{t}-d}_{e}}{d_{t}}\right)$$ (1)

where *d*_*e*_ is the diameter of each strand in 3D-printed scaffolds and *d*_*t*_ is the diameter of each strand in the designed one. However, the printability of the hydrogels is usually greater than one due to their high degree of swelling. Each experiment was repeated three times. To find the statistical difference between the 3D-printed samples compared to the control; first, the Gaussian distribution of the results was confirmed with Lavene test and then the results were analyzed with one-way ANOVA test.

Additionally, the percentage of coefficient of variation (CV%) was calculated for each strand in each sample.

### 2.6 Morphology of 3D-printed scaffolds

The microstructure and morphology of wet and dried 3D-printed samples were visualized using a field-emission scanning electron microscope (FE-SEM), and synchrotron radiation propagation-based imaging computed tomography (SR-PBI-CT) given its promising results for visualization and characterization of scaffolds, especially hydrogels (Izadifar et al., 2016; Izadifar et al., 2017b; Duan et al., 2021; Ning et al., 2021).

The 2D images of the 3D-printed scaffolds after lyophilization were captured with FE-SEM (Hitachi SU8010, Japan).

The SR-PBI-CT imaging experiments were performed at the Biomedical Imaging and Therapy 05ID-2 beamline (Wysokinski et al., 2007), Canadian Light Source (CLS), Canada. SR-PBI-CT, a synchrotron phase-based imaging technique, has the great advantage of simple implementation and faster acquisition with seconds to minutes compared to Magnetic Resonance Imaging (MRI). Benefiting from the high coherence of X-ray, SR-PBI-CT can achieve a high-contrast imaging for low-density scaffolds, especially when combined with phase retrieval (PhR) (Ning et al., 2020). These two characteristics make this technique a great tool for visualizing soft tissues without any tissue processing (Ning et al., 2020). The scans were performed while the scaffolds were placed in a tube filled with waters. All the scans were performed at a distance from the sample to the detector (SDD) of 1.5 m and a photon energy of 30 keV, which can provide satisfactory contrast and spatial resolution for imaging hydrogel scaffolds. For obtaining high-quality CT images with high contrast and low noise, a popular phase retrieval algorithm, transport-of-intensity (TIE) (Paganin et al., 2002), was performed on each projection with a δ/β value of 1,000. An open-source software package (the Ultra-Fast-Online, UFO) was used to perform PhR (i.e., TIE) on the projections and the CT reconstruction (filtered-back projection (FBP) algorithm) (Vogelgesang et al., 2016). The 3D data of scaffolds were then partially segmented at the center of the printed scaffolds using a 3D Slicer software (Fedorov et al., 2012).

### 2.7 Physical properties

To have a successful myocardial tissue regeneration, scaffolds encapsulating cardiomyocytes should be degradable allowing the cardiomyocytes to deposit their extracellular matrix to form aligned fibers (Black et al., 2009; Camci-Unal et al., 2014). The swelling of the scaffolds exposes them to hydrolytic degradation, which also can generate wall stress to the surrounding tissue. As a result, an existing balance between degradation and the swelling rate is required when implanting a scaffold inside the body (Saravanan et al., 2018) Also, due to the highly dynamic environment of the cardiac tissue, elastic materials (such as hydrogels) can be a better match for the tissue compared to non-elastic materials (Camci-Unal et al., 2014). Here, the physical properties of the 3D-printed scaffolds were studied by measuring their elastic modulus, swelling and mass loss percentage.

The elastic modulus is a measure of an object’s resistance to deformation in response to an applied force. Unlike stiffness, this measure is independent of the object geometry, allowing for comparison of the elastic modulus of samples with different shapes and sizes (Emig et al., 2021). For cells to adhere to a substrate and migrate, they need to generate traction stresses against the substrate. These forces are also used by cells to sense the substrate (Califano and Reinhart-King, 2010). It has been found that cells can behave differently based on the stiffness of a substrate that they inhabit (Hadden et al., 2017; Balcioglu et al., 2020; Körner et al., 2021). To measure the elastic modulus of the 3D scaffolds, compression force was applied using Bose BioDynamic ™ device (with a 20N load cell) to record stress-strain curves. The scaffolds were compressed to 50% of their initial height at a speed of 0.01 mm/s. The slope of the linear region of stress-strain curves was calculated as the elastic modulus of each scaffold.

To assess the swelling and mass loss characteristics of 3D-printed scaffolds, they were maintained in a complete culture media at 37°C and 5% CO2. The scaffolds were 3D-printed and kept under the sterile condition to avoid any contaminations, which may affect the swelling and mass loss percentage of the scaffolds. Following 3D printing, the scaffolds were removed, blotted with Kimwipes™, and weighed to record the initial weight of the scaffolds (W_0_). Then they were maintained in cell culture media and incubated at 37°C with 5% CO_2_. The wet weight of the scaffolds (W_t_) was then measured after 3, 7, 14, and 21 days of incubation to calculate the swelling percentage of the scaffolds using the formula as follows:

$$Swelling\;\left(\%\right)=\frac{W_{t}-W_{0}}{W_{0}}\times 100$$ (2)

where *W*_*t*_ is the wet weight of the scaffolds at different time points and *W*_0_ is the initial wet weight of the scaffolds after 3D-printing. For each time point, the swelling percentage was calculated for five replicates.

After weighing each scaffold at days 3, 7, 14, and 21 for the swelling test, the same scaffolds were freeze-dried to measure the dried mass at specific time points (*W*_*t*_). For initial lyophilized mass, separate scaffolds were 3D-printed, and their mean weight was considered as initial lyophilized mass. The mass loss percentage of the scaffolds was obtained using the formula (Sarker et al., 2019):

$$Mass\;loss\;\left(\%\right)=\frac{W_{0}-W_{l}}{W_{0}}\times 100$$ (3)

where *W*_*0*_ is the initial lyophilized weight and *W*_*l*_ is the lyophilized weight of the scaffolds at different time points.

### 2.8 Cell viability for 3D-printed samples

Following the 3D-printing of the scaffolds, they were incubated at 37°C with 5% CO_2_ overnight. The next day, the culture media was completely removed from the well and 100 µL of cell suspension (0.8 × 10^5^ cells) was seeded onto each scaffold. After 2 h, when the cells started attaching to the surface, more culture media was added to each scaffold and incubated for 48 h. For each group and cell type, five replicates were prepared.

At 48 h after seeding, the viability of cells was studied by MTT assay. In MTT assays, the metabolic activity of the viable cells is measured through mitochondrial metabolic rate, providing the number of viable cells indirectly (Rai et al., 2018). The assay was repeated two times, each performed in three replicates (n = 6). To perform the MTT assay, the old media was removed and 1.5 mL of MTT in fresh media (0.1 mg/mL) was added to each well. After 2 hours of incubation at 37 °C, the media was removed. To distinguish between the cells grown on the scaffolds from the cells grown around them or on the surface of the plate, the scaffolds were removed from the plates and placed into a new plate. Then 1.5 mL of DMSO was added to both the plates that the scaffolds were removed from and to the new plates with the scaffolds inside them. 2D cell monolayer was used as a control group. The plates were then placed on a shaker (300 rpm) for 2 h and the absorbance was read at wavelength of 550 nm using a BioTek Synergy HT Multi-Detection Microplate Reader.

To visualize the viability of the cells, the scaffolds were also stained with calcein Am and PI after 48 h of seeding the cells as previously described in Section 2.3.

### 2.9 Statistics

All quantitative data are shown as the means ± standard deviation. Statistical analyses were performed with one-way ANOVA using GraphPad Prism version 9.3.1. Legend **** on figures represents *p* < 0.0001, *** represents *p* < 0.001, ** represents *p* < 0.01, * represents *p* < 0.05, and ns represents non-significant.

## 3 Results and discussion

### 3.1 Cell viability on 2D alginate and gelatin hydrogels

**FIGURE 2:**
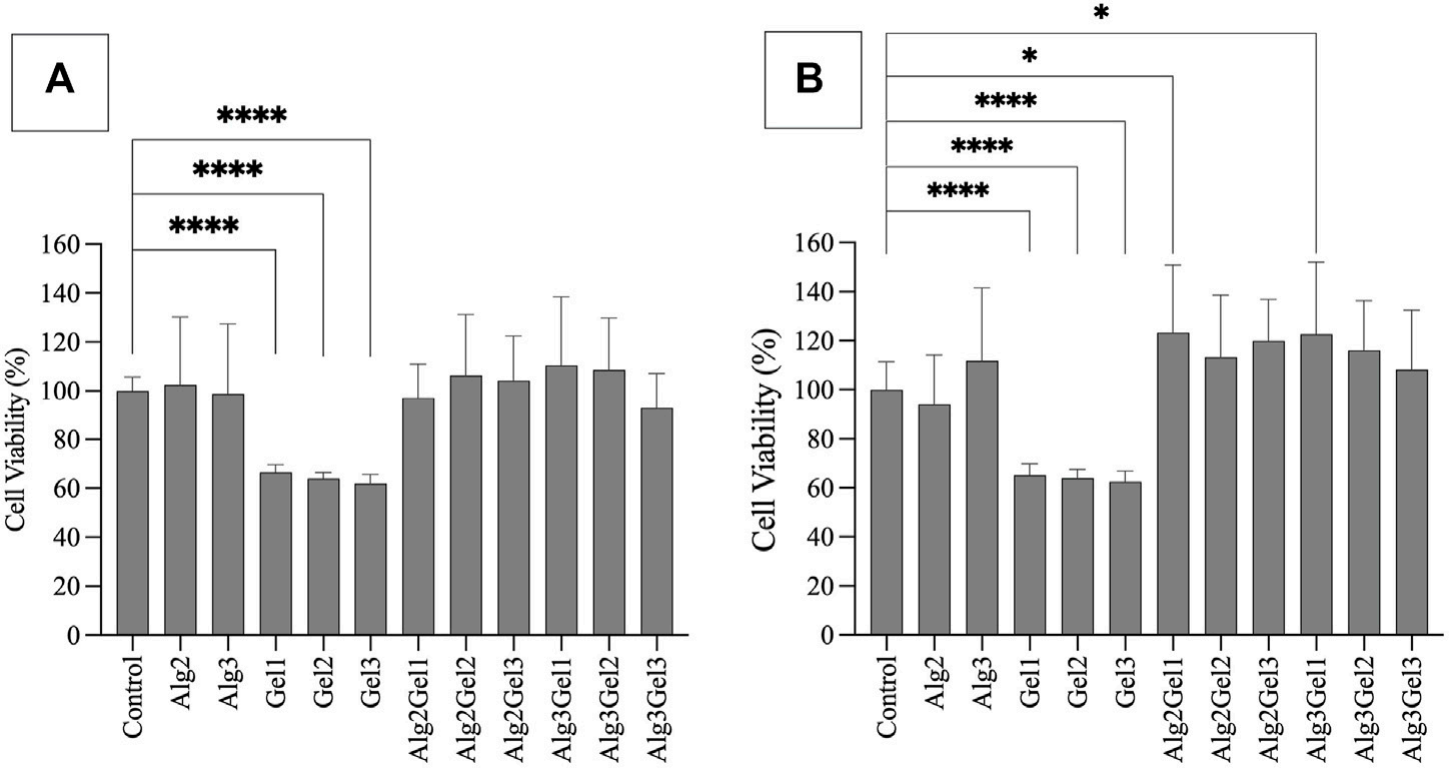
Cell viability of H9c2 cells **(A)** and HUVECs **(B)** using MTT assay. Cells (8,000 cells/well) were seeded on hydrogel coated 96 well-plates. The MTT was added 48 h after cell seeding and their absorbance was read at 550 nm. The cell viability percentage was normalized with cells only as the control. The results were compared together and to the control with One-way ANOVA followed by *post hoc* Dunnett test. The statistical difference between viability of cells seeded on the hydrogels compared to the control is shown with different numbers of asterisks.

Based on MTT assay, the viability of H9c2 cells seeded onto Alg2, Alg3, and the combinations of Alg and Gel were all more than 92%, showing no statistically significant differences. However, Gel1-Gel3 indicated significantly less viability (66.50%–61.91%) compared to the control and other hydrogels (Figure 2A). The viability of HUVECs on Alg2Gel1 (123.3%) and Alg3Gel1 (122.6%) showed statistically higher cell viability, indicating higher proliferation rate in these samples compared to the controls (Figure 2B).

**FIGURE 3:**
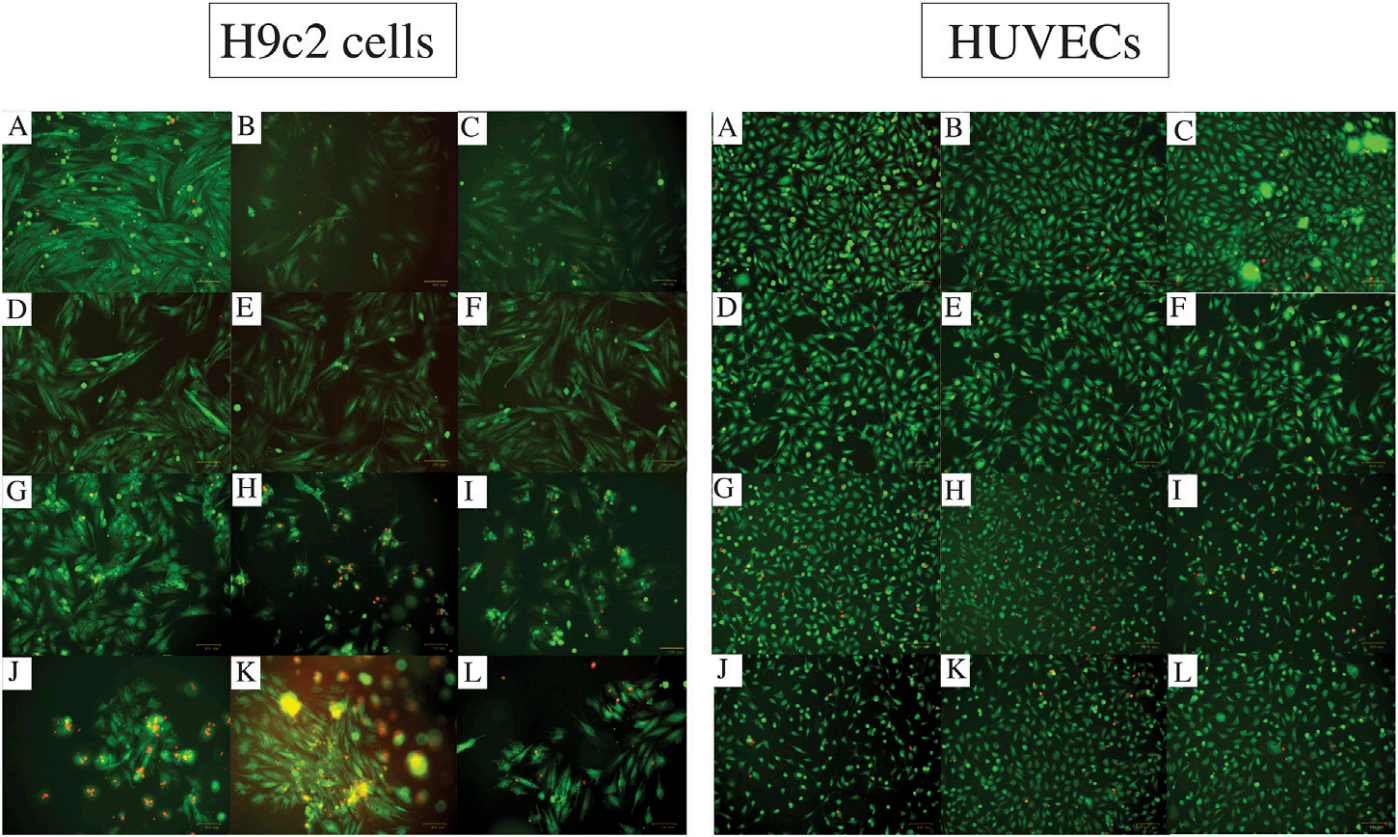
Cell viability assessment with Live/dead assay for different combinations of alginate and gelatin hydrogel 48 h after seeding H9c2 cells (the left images **(A-L)**) and HUVECs (the right images **(A-L)**). The live cells are stained with Calcein Am (green fluorescence) and dead cells are dyed with PI (red fluorescence). Images A show the cells without any hydrogel coating as controls. Images **(B)** and **(C)** show Alg2 and Alg3, respectively. Images **(D)** to **(F)** represent Gel1-3. The rest of the images show different combinations of Alg and Gel, including **(G)** Alg2Gel1, **(H)** Alg2Gel2, **(I)** Alg2Gel3, **(J)** Alg3Gel1, **(K)** Alg3Gel2, **(L)** Alg3Gel3.

The images captured from live/dead assay (Figure 3) confirm high cell viability in all the combinations. Both H9c2 cells and HUVECs show a similar phenotype to their control (no hydrogel-coating) following attaching to the hydrogel-coated plates. This applies to all the single and composite hydrogels. Although the majority of H9c2 cells and HUVECs seeded onto the gelatin alone are viable and show the expected phenotype, Gel 1%–3% did not enhance cell proliferation. However, combining Alg2 with Gel1 and Alg3 with Gel1 display high cell viability in addition to higher number of cells, indicating cell proliferation for HUVECs. Similar results are observed for H9c2 cells except for Alg3Gel1, showing few dead cells despite high cell viability.

As a result, Alg2Gel1 and Alg3Gel1 were selected for further experiments.

### 3.2 Strand printability evaluation

The strand printability of the Alg2Gel1 and Alg3Gel1, 3D-printed with various strand diameters and infill distances for both lattice and angular structures is calculated and compared to the ideal printability (=1) (Table 1). Alg2Gel1[1.5,0.2]-L and Alg2Gel1[1,0.2]-A were the most printable structures among two different structures constructed from Alg2Gel1 with the printability of 1.50 and 1.93, respectively. For Alg3Gel1 scaffolds, Alg3Gel1[1.5,0.2]-L, Alg3Gel1[1.5,0.2]-A, and Alg3Gel1[1.3,0.2]-A with the printability values of 1.41, 1.28 and 1.28 showed the highest values, indicating the lowest printability among Alg3Gel1 scaffolds.

**TABLE 1:** The strand printability of Alg2Gel1 and Alg3Gel1 scaffolds. Statistical differences were calculated using One sample *t*-test while each printability value was compared to a hypothetical value (=1).

Lattice scaffolds	Printability	Statistical difference	Angular scaffolds	Printability	Statistical difference
Alg2Gel1[1.5,0.2]-L	1.50 ± 0.32	ns	Alg2Gel1[1.5,0.2]-A	2.10 ± 0.06	**
Alg2Gel1[1.5,0.15]-L	2.96 ± 0.19	**	Alg2Gel1[1.5,0.15]-A	2.33 ± 0.52	*
Alg2Gel1[1.5,0.1]-L	4.05 ± 0.39	**	Alg2Gel1[1.5,0.1]-A	4.20 ± 0.33	**
Alg2Gel1[1.3,0.2]-L	2.14 ± 0.07	**	Alg2Gel1[1.3,0.2]-A	2.13 ± 0.25	*
Alg2Gel1[1.3,0.15]-L	2.40 ± 0.38	*	Alg2Gel1[1.3,0.15]-A	2.76 ± 0.09	***
Alg2Gel1[1.3,0.1]-L	4.00 ± 0.88	*	Alg2Gel1[1.3,0.1]-A	4.00 ± 0.39	**
Alg2Gel1[1,0.2]-L	1.66 ± 0.13	*	Alg2Gel1[1,0.2]-A	1.93 ± 0.09	**
Alg2Gel1[1,0.15]-L	2.64 ± 0.82	ns	Alg2Gel1[1,0.15]-A	2.51 ± 0.18	**
Alg2Gel1[1,0.1]-L	2.11 ± 0.02	***	Alg2Gel1[1,0.1]-A	3.86 ± 0.37	**
Alg3Gel1[1.5,0.2]-L	1.41 ± 0.16	*	Alg3Gel1[1.5,0.2]-A	1.28 ± 0.04	**
Alg3Gel1[1.5,0.15]-L	2.00 ± 0.10	**	Alg3Gel1[1.5,0.15]-A	1.82 ± 0.04	***
Alg3Gel1[1.5,0.1]-L	3.10 ± 0.09	***	Alg3Gel1[1.5,0.1]-A	2.48 ± 0.07	***
Alg3Gel1[1.3,0.2]-L	1.44 ± 0.11	*	Alg3Gel1[1.3,0.2]-A	1.28 ± 0.01	***
Alg3Gel1[1.3,0.15]-L	2.00 ± 0.05	***	Alg3Gel1[1.3,0.15]-A	1.50 ± 0.09	**
Alg3Gel1[1.3,0.1]-L	3.12 ± 0.25	**	Alg3Gel1[1.3,0.1]-A	2.46 ± 0.03	***
Alg3Gel1[1,0.2]-L	1.52 ± 0.02	***	Alg3Gel1[1,0.2]-A	1.31 ± 0.19	ns
Alg3Gel1[1,0.15]-L	1.74 ± 0.17	*	Alg3Gel1[1,0.15]-A	2.16 ± 0.21	*
Alg3Gel1[1,0.1]-L	3.00 ± 0.10	***	Alg3Gel1[1,0.1]-A	3.24 ± 0.27	**

Structures with a strand diameter of 0.1 mm showed the highest deviation from the theoretical value, causing the highest values of printability. Conversely, samples with the highest strand diameter of 0.2 mm mostly showed lower values of printability regardless of the infill distances. Following analyzing the values of printability for each sample, the printability of majority of the scaffolds worsened by decreasing the strand diameter in samples with same infill distances (one-way ANOVA, followed by Holm-Šídák’s multiple comparisons test, Supplementary Table S1). These results suggest that the difference between theoretical and angular strand diameters with the same or different infill distances (ranging from 1 mm to 1.5 mm) might increase with decreasing the strand diameter from 0.2 mm to 0.1 mm in majority of the samples. Therefore, the printability of the strands was only affected by strand diameter, and different infill distances did not show any effects on the strand printability.

**FIGURE 4:**
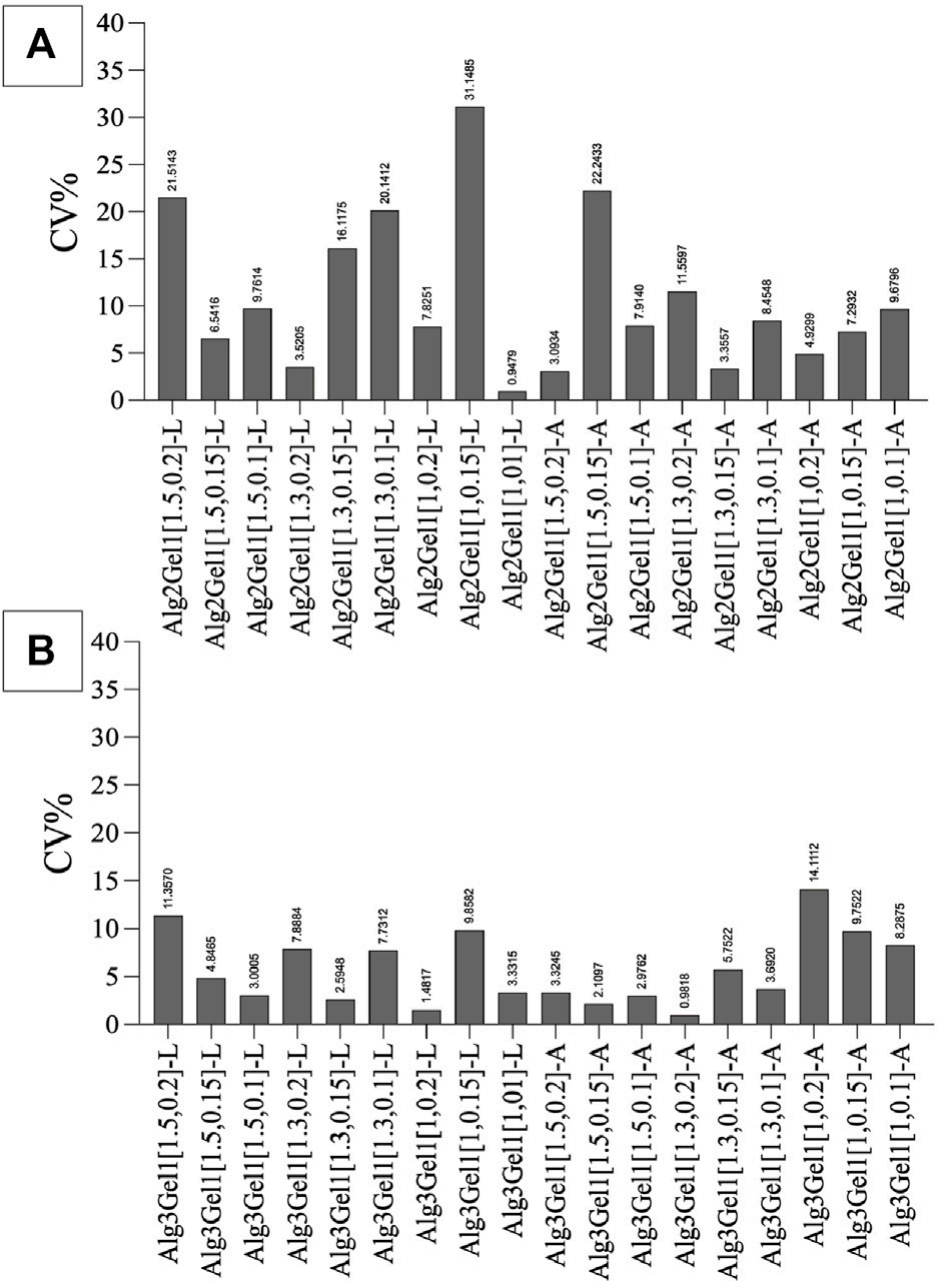
The coefficient of variation (CV%) of strand diameters in Alg2Gel1 **(A)** and Alg3Gel1 **(B)**. Following printing two layers of each structure with various strand diameters and infill distances, the difference between the diameters of theoretical and 3D-printed strands was measured.

To narrow down the structures based on their reproducibility, the CV% of the strands was calculated for each structure. Alg2Gel1[1,0.1]-L, Alg2Gel1[1,0.1]-A, Alg2Gel1[1,0.2]-L and Alg2Gel1[1,0.2]-A with CV% less than 10% were selected as the Alg2Gel1 structures with the highest reproducibility (Figure 4A). Among structures fabricated from Alg3Gel1, Alg3Gel1[1.5,0.15]-L, Alg3Gel1[1.5,0.15]-A, Alg3Gel1[1.5,0.1]-L, and Alg3Gel1[1.5,0.1]-A with CV% less than 5% were selected for further studies (Figure 4B).

### 3.3 Morphology and scaffolds visualization

**FIGURE 5:**
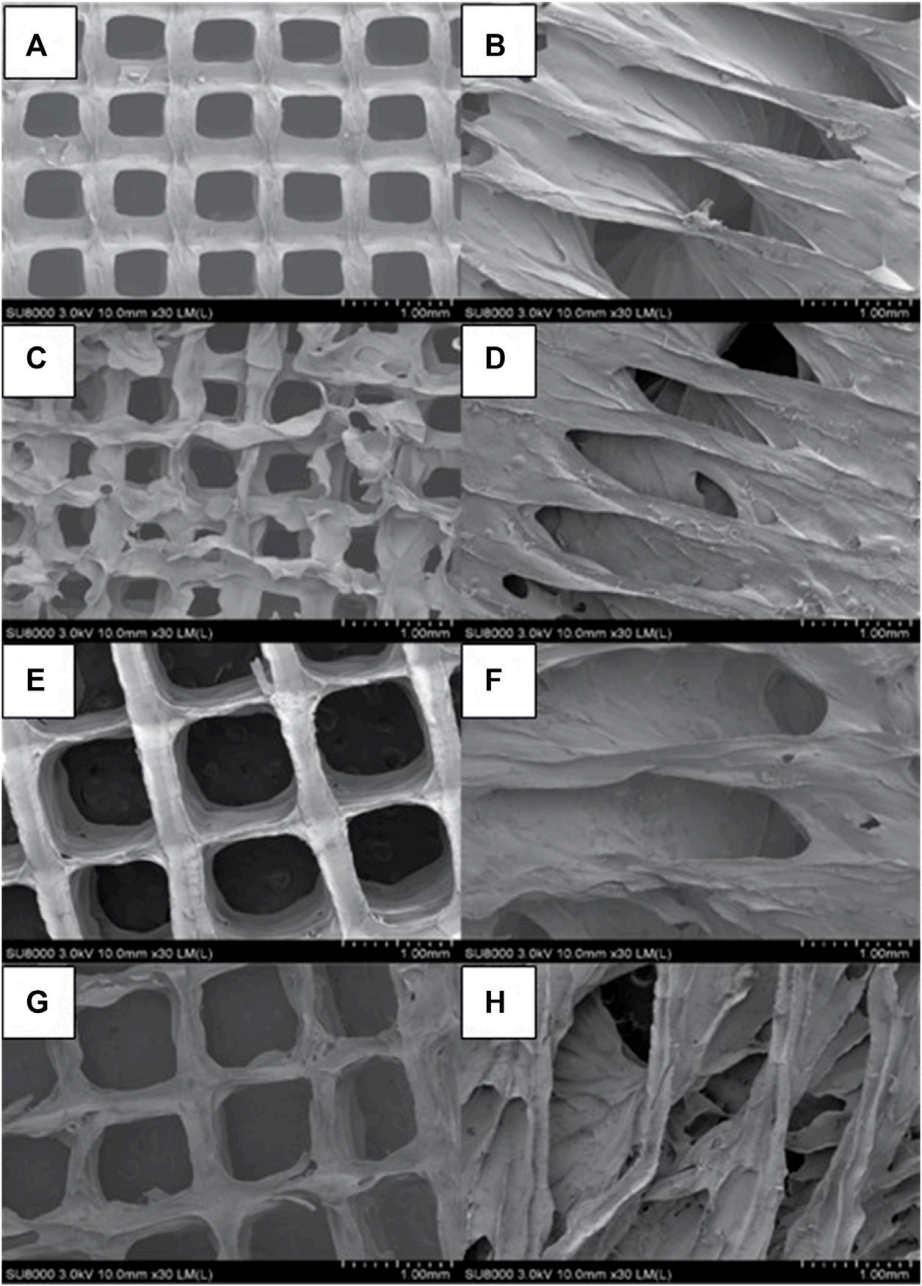
FE-SEM images of lyophilized 3D-printed scaffolds **(A)** Alg2Gel1[1,0.2]-L, **(B)** Alg2Gel1[1,0.2]-A, **(C)** Alg2Gel1[1,0.1]-L, **(D)** Alg2Gel1[1,0.1]-A, **(E)** Alg3Gel1[1.5,0.15]-L, **(F)** Alg3Gel1[1.5,0.15]-A, **(G)** Alg3Gel1[1.5,0.1]-L, **(H)** Alg3Gel1[1.5,0.1]-A.

The two-dimensional (2D) and lyophilized structures constructed from Alg2Gel1 and Alg3Gel1 were captured using FE-SEM (Figure 5). The images for Alg2Gel1[1,0.2]-L (Figure 5A) and Alg2Gel1[1,0.2]-A (Figure 5B) indicate a uniform structure within 15 layers. However, Alg2Gel1[1,0.1]-L (Figure 5C) does not show a consistent lattice structure compared to the other groups. Although Alg2Gel1[1, 0.1]-A is uniform (Figure 5D), the structure of this group collapsed frequently after 3D-printing of about 10–12 layers. As a result, Alg2Gel1[1,0.1]-L and Alg2Gel1[1, 0.1]-A were eliminated from the study. The images for Alg3Gel1[1.5,0.15]-L (Figure 5E), Alg3Gel1[1.5,0.15]-A (Figure 5F), and Alg3Gel1[1.5, 0.1]-L (Figure 5G), and Alg3Gel1[1.5,0.1]-A (Figure 5H) all presented a uniformity throughout the layers.

**FIGURE 6:**
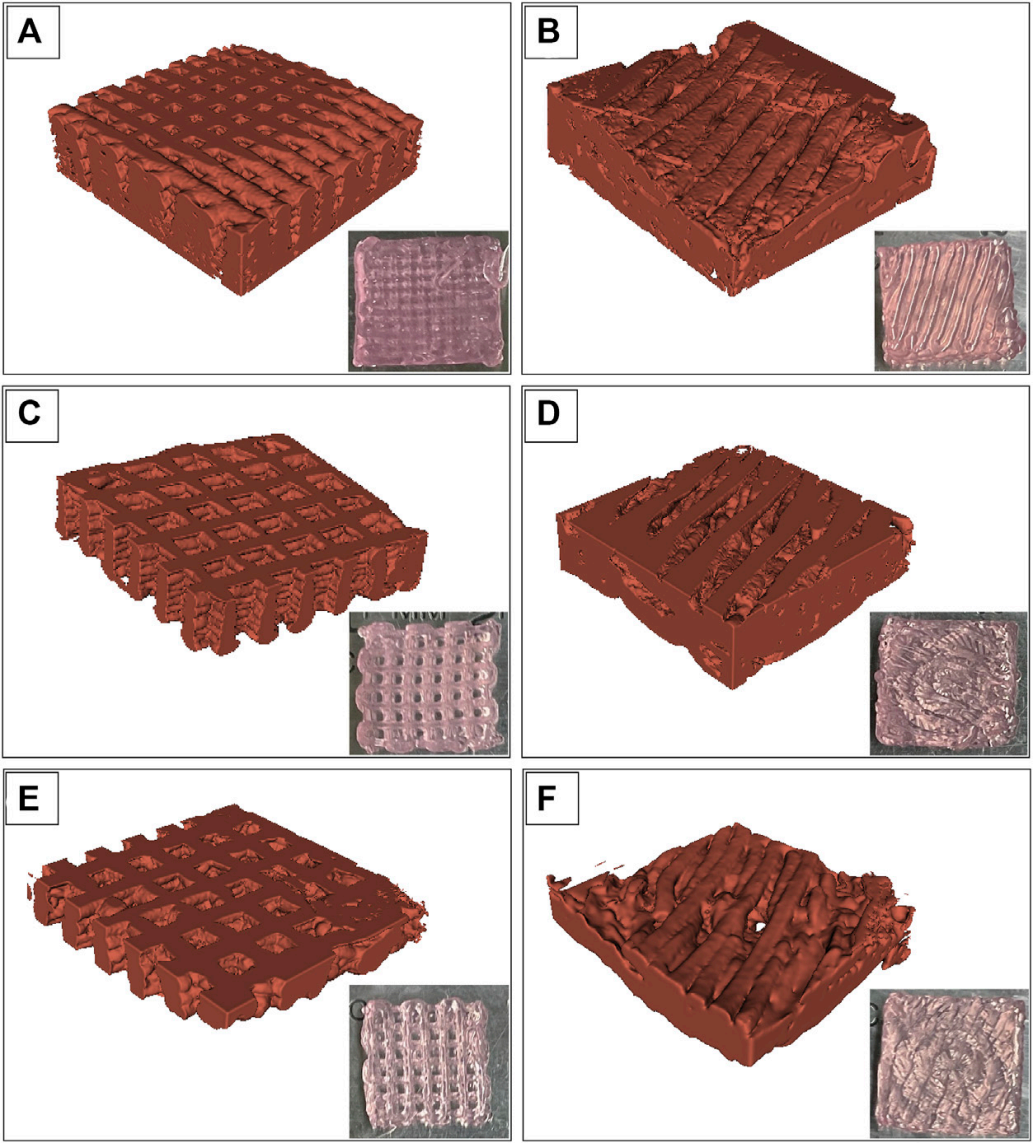
Images from swollen scaffolds with camera *versus* reconstructed images captured by synchrotron radiation propagation-based imaging computed tomography (SR-PBI-CT) **(A)** Alg2Gel1[1,0.2]-L, **(B)** Alg2Gel1[1,0.2]-A, **(C)** Alg3Gel1[1.5,0.15]-L, **(D)** Alg3Gel1[1.5,0.15]-A, **(E)** Alg3Gel1[1.5,0.1]-L, **(F)** Alg3Gel1[1.5,0.1]-A.

The images of the Alg2Gel1 and Alg3Gel1 scaffolds while they were soaking in water were captured using SR-PBI-CT and segmented partially with a 3D slicer. Figure 6 shows a rebuilt 3D structure of the scaffolds and the 2D images taken by a camera. The 3D structures of the Alg2Gel1[1, 0.2]-L (Figure 6A **)**, Alg2Gel1[1, 0.2]-A (Figure 6B), Alg3Gel1[1.5, 0.15]-L (Figure 6C), Alg3Gel1[1.5, 0.15]-A (Figure 6D), and Alg3Gel1[1.5, 0.1]-L (Figure 6E), Alg3Gel1[1.5, 0.1]-A (Figure 6F) are all relatively uniform.

The strand diameter and pore area were measured on each image captured by FE-SEM and SR-PBI-CT (Table 2). For most of the scaffolds imaged by SR-PBI-CT, the strand diameter was larger compared to the images from FE-SEM since the scaffolds were swollen and soaked in water before and during the imaging. The images captured from angular scaffolds with FE-SEM present different pores in various ranges of sizes due to their design. However, the pores in angular samples from SR-PBI-CT are more uniform due to the non-invasive nature of this imaging technique that does not need any sample processing, such as cutting or desiccation.

**TABLE 2:** Strand diameter and pore area of the 3D printed scaffolds, measured from FE-SEM and SR-PBI-CT images.

3D printed scaffolds	FE-SEM		SR-PBI-CT	
	Strand diameter (mm)	Pore area (mm)	Strand diameter (mm)	Pore area (mm)
Alg2Gel1[1,0.2]-L	0.269 ± 0.038	0.223 ± 0.024	0.328 ± 0.076	0.141 ± 0.029
Alg2Gel1[1,0.2]-A	0.173 ± 0.036	0.055 ± 0.012 to 0.927 ± 0.236	0.370 ± 0.054	0.399 ± 0.134
Alg2Gel1[1,0.1]-L	0.23 ± 0.064	0.119 ± 0.025	-	-
Alg2Gel1[1,0.1]-A	0.214 ± 0.027	0.024 ± 0.014 to 0.704 ± 0.187	-	-
Alg3Gel1[1.5,0.15]-L	0.079 ± 0.013 to 0.264 ± 0.022	0.860 ± 0.240	0.159 ± 0.029	0.143 ± 0.027
Alg3Gel1[1.5,0.15]-A	0.210 ± 0.048	0.022 ± 0.012 to 2.184 ± 0.343	0.260 ± 0.032	0.191 ± 0.082 to 1.217 ± 0.291
Alg3Gel1[1.5,0.1]-L	0.228 ± 0.048	0.712 ± 0.056	0.370 ± 0.082	0.305 ± 0.076
Alg3Gel1[1.5,0.1]-A	0.181 ± 0.038	0.041 ± 0.010 to 0.798 ± 0.389	0.382 ± 0.048	0.749 ± 0.122

### 3.4 Physical properties of the scaffolds

**FIGURE 7:**
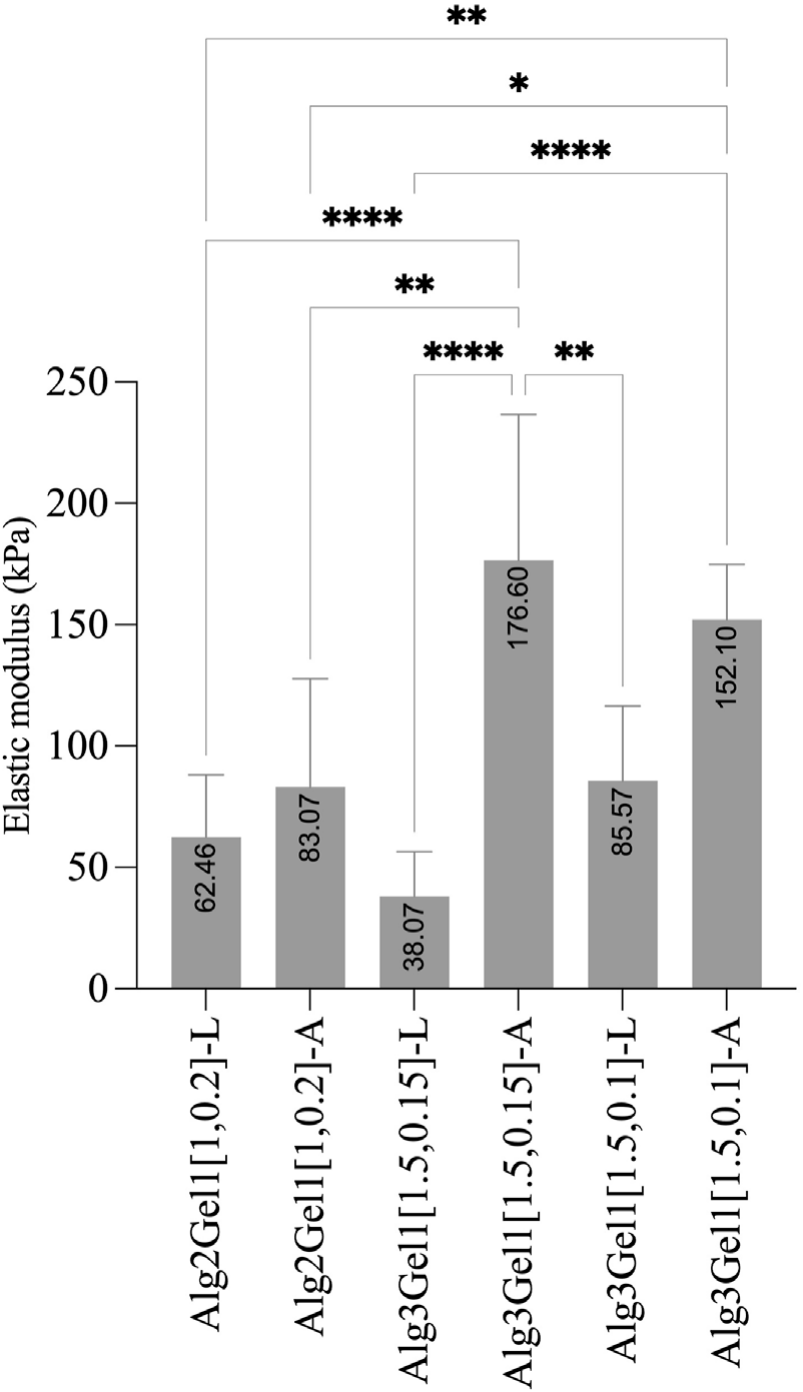
Elastic modulus of Alg2Gel1 and Alg3Gel1 for both lattice and angular structures. The mean value of each group was compared with the mean of other groups with one-way ANOVA followed by *post hoc* Tukey’s test.

The elastic modulus of the 3D scaffolds on the day of 3D-printing is shown in Figure 7. The elastic modulus of Alg2Gel1[1,0.2]-L and Alg2Gel1[1,0.2]-A was measured as 62.46 ± 25.67 kPa and 83.07 ± 44.71 kPa, respectively. The elastic modulus of Alg3Gel1[1.5,0.15]-L, Alg3Gel1[1.5,0.15]-A, Alg3Gel1[1.5,0.1]-L, and Alg3Gel1[1.5,0.1]-A were 38.07 ± 18.37, 176.6 ± 60.05, 85.57 ± 30.99, and 152.10 ± 22.7 kPa, respectively. The elastic modulus of angular structures increased compared to the lattice scaffolds for both Alg2Gel1 and Alg3Gel1. In Alg2Gel1 scaffolds, the elastic modulus of lattice *versus* angular does not show any statistically significant differences, however, there is a statistical difference in the Alg3Gel1 groups.

**FIGURE 8:**
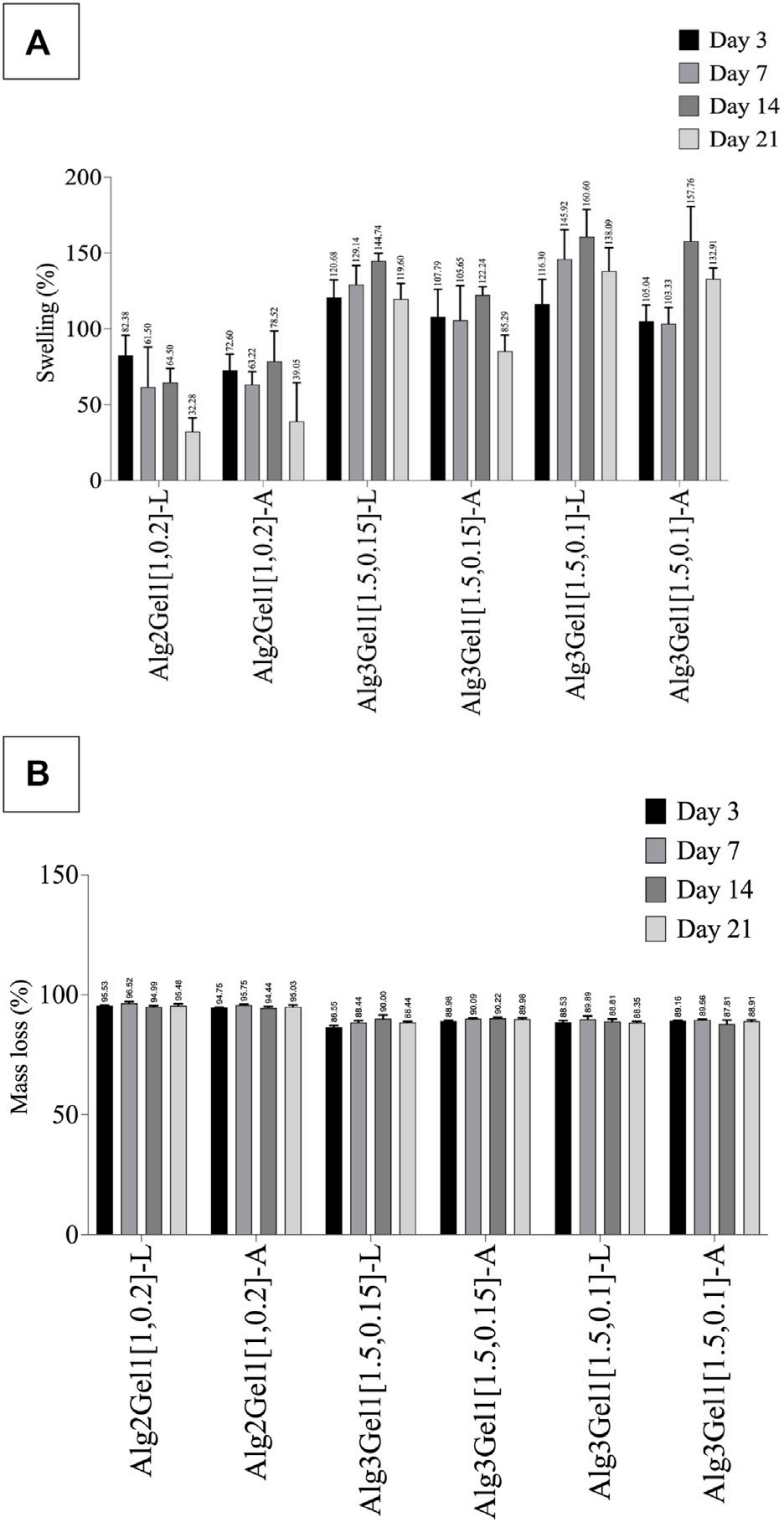
Swelling percentage **(A)** and mass loss percentage **(B)** of Alg2Gel1 and Alg3Gel1 scaffolds during 21 days of incubation in cultre media at 37°C. The data were compared together with one-way ANOVA followed by *post hoc* Tukey’s test. The statistical significance among the groups are discussed in the text.

The effect of incubation time on swelling percentage of the 3D scaffolds has been presented in Figure 8A.

All the scaffolds composed of Alg3Gel1 reached a swelling percentage between 105.03% (Alg3Gel1[1.5,0.1]-A) to 120.68% (Alg3Gel1[1.5,0.15]-L) by day 3. These scaffolds continued to swell more gradually, reaching their maximum swelling on day 14. The swelling of Alg3Gel1 started to decline from day 21. At each time point, the angular scaffolds in each group showed less swelling compared to the lattice structures.

Scaffolds made of Alg2Gel1 showed a lower swelling percentage compared to Alg3Gel1 during the period of the experiment. They showed a swelling percentage between 72. 60% (angular) to 82.37% (lattice) by day 3. The swelling percentage of Alg2Gel1 scaffolds started to fluctuate by day 14 and then reached 32.28% for lattice and 39.04% for angular scaffolds on day 21. Starting from day 7, all the Alg3Gel1 scaffolds presented statistically higher swelling percentages compared to the Alg2Gel1 scaffolds. On day 7, the swelling percentage of Alg3Gel1[1.5,0.1]-L (145.92%) was statistically higher than the angular one (Alg3Gel1[1.5,0.1]-A, 103.33%, *p*-value: 0.0101). On day 21, Alg3Gel1[1.5,0.15]-A scaffolds showed less swelling percentage (85.29%) compared to the lattice one (Alg3Gel1[1.5,0.15]-L, 119.60%, *p*-value: 0.0124). There was also a significant difference between the swelling percentage of Alg3Gel1[1.5,0.15]-A (85.29%) compared to Alg3Gel1[1.5,0.1]-A (132.90%, *p*-value: 0.0003). No significant differences were seen between lattice and angular structures of Alg2Gel1.

The mass loss percentage of 3D-printed scaffolds has been shown in Figure 8B. All the scaffolds lost more than 86% of their initial weight after 3 days of incubation in culture media. However, the mass loss percentage of all the groups remained constant or very slightly increased for the rest of the experimental period. Alg2Gel1 scaffolds showed a significantly higher mass loss percentage than the Alg3Gel1 groups at each time point (*p*-value <0.0001). After 21 days of incubation, Alg3Gel1 scaffolds showed a mass loss percentage of less than 90%, while Alg2Gel1 scaffolds lost more than 95% of their initial weight.

### 3.5 Cell viability for 3D-printed structures

**FIGURE 9:**
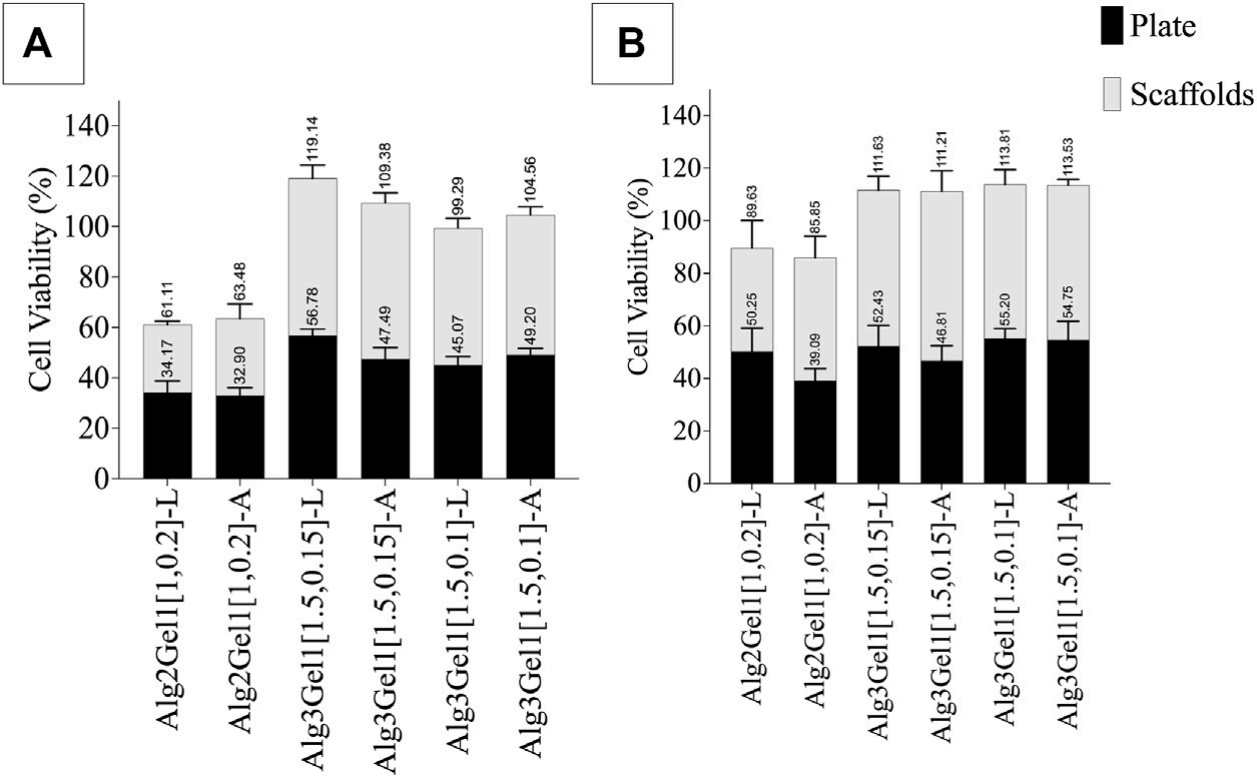
Viability of **(A)** H9c2 cells and **(B)** HUVECs 48 h after seeding 8,000 cell on each 3D-printed scaffold. The viability of the cells on the scaffolds and plate was quantified separately. Multiple comparisons among the groups including the viability of the cells on the plate verus scaffolds were performed by one-way ANOVA followed by *post hoc*Sidak’s test.

**FIGURE 10:**
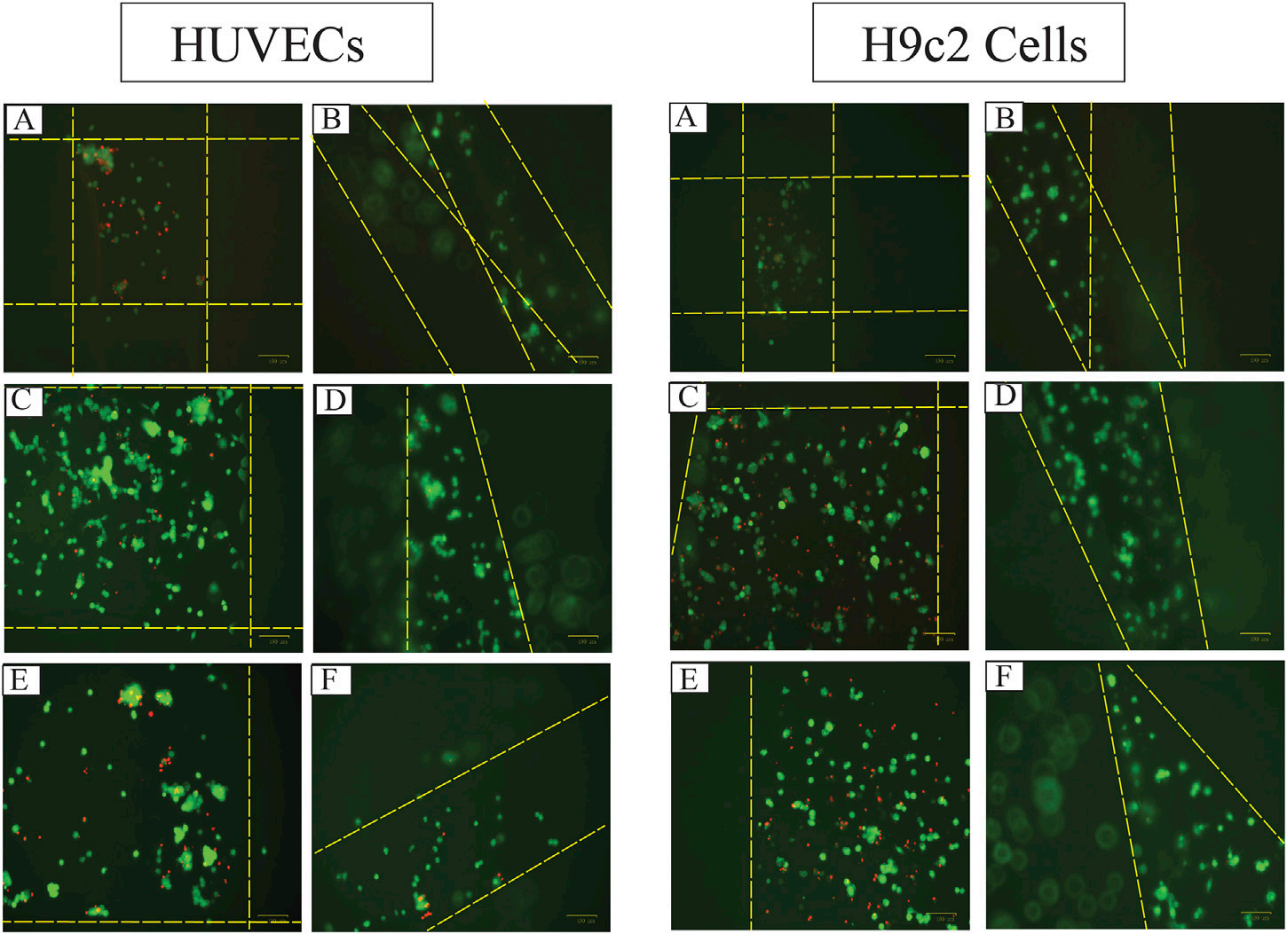
The fluorescent images of live (green) and dead (red) HUVECs and H9c2 cells 48 h after seeding 8,000 cells on 3D-printed **(A)** Alg2Gel1[1,0.2]-L, **(B)** Alg2Gel1[1, 0.2]-A, **(C)** Alg3Gel1[1.5,0.15]-L, **(D)** Alg3Gel1[1.5,0.15]-A, **(E)** Alg3Gel1[1.5,0.1]-L, **(F)** Alg3Gel1[1.5,0.1]-A scaffolds (the dotted yellow lines are the location of the strands).

The viability of H9c2 cells and HUVECs was quantitatively and qualitatively studied through MTT (Figure 9) and live/dead assays (Figure 10), respectively. To account for the cells that attached directly onto the cell culture plates instead of remaining on the scaffold, MTT results are presented as the sum of cell viability on the scaffolds and cells that remained on the cell culture plate after removing the scaffolds.

The viability of HUVECs 2 days following seeding them onto Alg3Gel1 scaffolds show cell proliferation (viability over 100% compared to controls) and do not show any statistical differences between the two groups and the two structures. The viability of HUVECs on lattice scaffolds prepared with Alg2Gel1 are less than the cell viability of the same cells seeded on Alg3Gel1[1.5,0.15]-L and Alg3Gel1[1.5,0.1]-L scaffolds (*p* < 0.0005). Alg2Gel1[1,0.2]-A also showed less cell viability compared to Alg3Gel1[1.5,0.15]-A scaffolds (*p* = 0.0023). In all samples, the number of viable cells attached to the surface of the plate and the cells attached to the scaffolds after cell seeding was similar except for Alg3Gel1[1.5,0.15]-A, showing higher cell viability on scaffolds compared to the plate.

The viability of H9c2 cells on Alg2Gel1[1,0.2]-L is less than the cell viability of both Alg3Gel1[1.5,0.15]-L and Alg3Gel1[1.5,0.1]-L (*p* < 0.0001). All the scaffolds made of Alg3Gel1 presented a cellular viability of more than 100%, except for Alg3Gel1[1.5,0.1]-L (99.29%). The Alg2Gel1[1,0.2]-L scaffolds presented less cell viability compared to Alg3Gel1[1.5,0.15]-L. The Alg3Gel1[1.5,0.15]-L scaffolds showed higher cell viability compared to Alg3Gel1[1.5,0.1]-L. Both Alg2Gel1[1,0.2]-L and AlgGel1[1,0.2]-A scaffolds showed significantly less cell viability compared to Alg3Gel1[1.5,0.15]-A and Alg3Gel1[1.5,0.1]-A (*p* < 0.0001). The viability of H9c2 cells seeded on both scaffolds and plates are equal for all the samples, except for Alg3Gel1[1.5,0.15]-A and Alg3Gel1[1.5,0.1]-L scaffolds where the viable cells attached to the scaffolds are significantly higher than the viable cells attached to the plate, two days after seeding the cells onto the scaffolds (*p* < 0.0001 and *p* = 0.00965, respectively).

Based on the presented result, Alg3Gel1[1.5,0.15]-A could maintain more viable cells onto the scaffolds than the plate compared to the other samples.

The images captured after adding a live/dead assay kit for H9c2 cells and HUVECs seeded on 3D-printed scaffolds are shown in Figure 10. Both H9c2 cells and HUVECs show high viability on the 3D-printed hydrogels; however, their phenotype looks different when cultured in 3D compared to the cells seeded in 2D on hydrogel-coated or tissue culture plates. There are fewer H9c2 cells grown inside the Alg2Gel1 scaffolds compared to the Alg3Gel1 scaffolds. In Alg3Gel1 scaffolds, there are more cells entrapped in the pores of the lattice structures compared to the angular scaffolds due to geometry of the pores. Although there are fewer H9c2 cells penetrating in the angular pores, no dead cells are seen inside these pores. There are a few dead H9c2 cells in Alg3Gel1[1.5,0.15]-L, but no dead cells are observed in Alg3Gel1[1.5,0.15]-A scaffolds.

There are fewer viable HUVECs grown inside the lattice structure of Alg2Gel1 scaffolds compared to the angular structure. The number of viable HUVECs grown inside the Alg3Gel1 scaffolds is higher compared to the ones inside the Alg2Gel1 scaffolds. The Alg3Gel1[1.5,0.15]-L and Alg3Gel1[1.5,0.15]-A scaffolds provided a better substrate for HUVECs’ adhesion compared to Alg3Gel1[1.5,0.1]-L and Alg3Gel1[1.5,0.1]-A scaffolds.

## 4 Discussion

A 3D-printed cardiac construct should be biocompatible, and bioprintable, promote cellular functions and show mechanical strength and elasticity that is desirable to the cells (Izadifar et al., 2018).

Alginate/gelatin hydrogels are suitable materials for 3D-printed tissue constructs because a degree of tissue maturation can occur before the hydrogel disintegrates (Bociaga et al., 2019; Roche et al., 2021). Alginate is mainly used due to its mechanical stability and gelatin is utilized because it contains arginine-glycine-aspartic acid (RGD) sequence that is vital for a stable relationship between the cells and the surrounding ECM and beneficial for cell adhesion (Onoe et al., 2013; Kim et al., 2017). In addition to the nature of the materials, the viscosity of the prepared hydrogels is also important. On one hand, cells experience higher shear stress due to high extrusion pressure when 3D-printed within a hydrogel with high viscosity (Mondal et al., 2019). On the other hand, polymer concentration is a crucial factor to achieve desired biophysical properties in polymer-based gels depending on the purpose of the study (Borries et al., 2020). Therefore, finding a balance between cell viability and mechanical behaviour of the hydrogels is challenging (Gregory et al., 2022). Here, a primary challenge was to find combinations of materials for 3D-printed constructs that are viscous enough to be extruded into accurate structures and have acceptable cell viability (>80–90%) to apply to cell-seeding and cell-laden 3D-printing studies. In 2D, alginate alone did not affect the viability of both H9c2 cells and HUVECs. Although the viability of H9c2 cells and HUVECs were adversely affected by gelatin alone, the viability of HUVECs for the combinations of Alg2Gel1 and Alg3Gel1 promoted cell proliferation compared to the controls. Similarly, encapsulation of HUVECs with alginate/gelatin blends increased their viability compared to the HUVECs grown on the tissue culture plate directly (Nemati et al., 2017). Another study revealed that the encapsulation of H9c2 cells with alginate/gelatin microspheres could increase cell viability while maintaining the cells' multipotentiality (Saberianpour et al., 2019). Although gelatin is known for its merits such as biological origin and biocompatibility, it was reported that HUVECs have a higher proliferation rate on the cross-linked heparin-gelatin fibers compared to pure gelatin fibrous scaffold (Wang et al., 2013). Therefore, the presence of blended gelatin may be more beneficial for cell survival compared to gelatin alone.

The strand printability of the two combinations under constant speed and pressure of the 3D-printing head shows that with increasing the strand diameter, the printability value is decreasing toward one, meaning that the 3D-printed structure is more similar to the theoretical value. The structures with a strand diameter of 0.2 mm showed lower values of printability while the highest values of printability belonged to the structures with a strand diameter of 0.1 mm. This shows that the structures with a strand diameter of 0.1 mm could be closer to the minimum printable feature of the 3D-printer compared to the two other tested strand diameters (0.15, 0.2 mm) (Wang et al., 2021). However, to narrow down the number of structures with different strand diameters and infill distances, designs with lower CV% in their strand diameter were selected for further studies. In this context, the CV% is a good measure of how consistent the strand diameter is over the various 3D-printing replications (Patterson et al., 2019). Therefore, Alg2Gel1[1,0.1]-A, Alg2Gel1[1,0.1]-L, Alg2Gel1[1,0.2]-A, Alg2Gel1[1,0.2]-L, Alg3Gel1[1.5,0.1]-A, Alg3Gel1[1.5, 0.1]-L, Alg3Gel1[1.5, 0.15]-A, and Alg3Gel1[1.5,0.15]-L were selected for 3D-printing structures.

The physical properties of the 3D-printed structures were studied by measuring their elastic modulus, swelling and mass loss percentage. In our study, the elastic modulus of the angular designs is higher than that of lattice designs. The higher elastic modulus in the angular designs might be due to more contact area between strands from adjacent layers (You et al., 2017). It was shown that elastic modulus increased significantly by changing the structure from a lattice (0°/90°) to an angular design (0°/45°) with the same strand spacing (You et al., 2017). We found that with increasing the concentration of Alg from 2% to 3%, the elastic modulus of the scaffolds was increased. Our results are consistent with a study (Larsen et al., 2015), in which the elastic modulus of injectable sodium alginate was nearly doubled by doubling its concentration (Larsen et al., 2015). The elastic modulus of 3D printed lattice scaffolds composed of various concentrations of Alg/Gel was reported to be in the range of 29.8 ± 2.49 to 48.0 ± 5.74 which is in accordance with our results for lattice structure (di Giuseppe et al., 2018). This might be due to the formation of more interchain complexes involving many guluronate moieties (G-blocks) among different alginate chains (called “egg-box structure) in combinations with a higher concentration of sodium alginate during crosslinking with Ca^2+^ ions (Bruchet and Melman, 2015; Larsen et al., 2015). In our study, the elastic modulus of the angular scaffolds containing Alg3 was nearly twice of angular scaffolds containing Alg2, likey showing the synergistic effects of the polymer concentration and the angular design on elastic modulus. Besides the smaller infill distances in the Alg2Gel1 scaffolds (1 mm) compared to Alg3Gel1 scaffolds (1.5 mm), there might be fewer voids to be filled with culture media solution due to fewer interchain moieties in these scaffolds. Therefore, Alg2Gel1 scaffolds showed the least swelling percentage among the scaffolds. However, Alg2Gel1 showed the highest mass loss at all time points compared to Alg3Gel1. This can be attributed to a smaller number of formed interchain moieties between G blocks and less chain density (Chung et al., 2013), causing less mechanical strength and a higher percentage of mass loss. Although alginate fibers gradually lose their integrity and start to degrade from the exchange reaction of Ca^2+^ and the cations such as Na^+^ in the culture media (Yeo and Kim, 2020), Alg3Gel1 scaffolds presented lower percentage of mass loss compared to Alg2Gel1. Sodium alginate is a hydrophilic polysaccharide (Rehman et al., 2019) and its higher amount in Alg3Gel1 structures could make these scaffolds more hydrophilic compared to Alg2Gel1. Thus, the higher swelling percentage of Alg3Gel1 scaffolds is likely due to their higher hydrophilicity as well as a greater number of interchain moieties, more chain density, and a higher number of crosslink bonds among alginate chains, forming more cavities for liquid entrapment. While there are huge cubical free spaces between the strands in the lattice structure, there are various pores with varied sizes distributed within the scaffolds; consequently, Alg3Gel1 angular structures showed less swelling percentage, but higher elastic modulus compared to the lattice structure. It is worth mentioning that there is a fluctuation in swelling behavior of some of the scaffolds, first reaching a high absorbency and then a decrease in swelling percentage. This is a well-known and common phenomenon in ionic hydrogel. The swelling capacity of ionic hydrogels declines when the ionic strength of the swelling medium is increased, causing a non-perfect anion-anion electrostatic repulsion leading to a reduced osmotic pressure (ionic pressure) difference between the scaffolds and the external solution (Bao et al., 2011; Shahi et al., 2017).

MTT and live/dead assays were used to study the survival rate of H9c2 cells and HUVECs. To have a more precise comparison between the viability of the cells in lattice *versus* angular scaffolds, the metabolic activity of viable cells on the scaffolds and the plate were studied separately in the MTT assay. Our findings from both assays indicate that cell survival on scaffolds made of Alg3Gel1 is higher than on Alg2Gel1 scaffolds. It seems that for most of the scaffolds, there are more viable cells on the angular scaffolds compared to the viable cells on the plates. Overall, the angular scaffolds made of Alg3Gel1 with a strand diameter of 0.15 mm and infill distance of 1.5 mm provided the highest number of viable cells on the scaffolds. The results from MTT assays indicate that the number of viable H9c2 cells and HUVECs for all the Alg3Gel1 samples is in the same range and more than 99%. However, based on the images from the live/dead assay, the number of dead cells in the lattice structure is visibly higher than the dead cells on the angular scaffolds. A similar pattern is also repeated for samples with Alg2Gel1 scaffolds, where the ratio of viable cells to dead cells is less in the angular compared to the lattice scaffolds. In the lattice structures, the cells are entrapped in isolated cubical areas among the pores while in the angular designs, they are distributed among the strands. The higher number of viable cells in angular structures could be due to greater contact areas between the layers, providing a better environment for cell signaling, and proliferation. These findings are in accordance with a study (Liu et al., 2015) investigating the role of the alignment of polyurethane fibers on annulus fibrosus-derived stem/progenitor cells. The results from that study show that although the survival percentage of the seeded cells on both aligned fibers and random fibers are similar, the cells seeded on aligned fibers were more elongated with better alignment and showed higher expression and matrix production of collagen I and aggrecan (Liu et al., 2015). In recent years, a few studies have used additive manufacturing methods such as 3D-printing, in which they included two or more cell types into their scaffolds to generate a functional cardiac construct with vasculogenesis for cardiac tissue repair (Gaebel et al., 2011; Zhang et al., 2016; Gao et al., 2017; Jang et al., 2017; Ong et al., 2017; Zhu et al., 2017; Arai et al., 2018; Maiullari et al., 2018b; Ma et al., 2019; Noor et al., 2019; Yeung et al., 2019; Tsukamoto et al., 2020). Few studies are focusing on the effect of patterning of the substrate on cellular functions for cardiac tissue engineering (Zong et al., 2005; Courtney et al., 2006; Rubbens et al., 2009; Engelmayr et al., 2002; Kai et al., 2011; Parrag et al., 2012; Tseng et al., 2014; Yu et al., 2014; Zhang et al., 2015; Liu et al., 2017; Hitscherich et al., 2018; Lei et al., 2019; Basara et al., 2022), mostly focusing on the effect of random *versus* aligned electrospun fibers on cellular behavior.

To our knowledge, there are no studies focusing on 3D-printing a novel structure mimicking the orientation of the heart fibers and studying the effects of this structure on both cardiac and endothelial cells. Further investigations are required to develop functional cardiac tissue, including co-culturing both cells for a longer period under a dynamic condition mimicking the physiological condition. Although H9c2 cells show many similarities to primary cardiomyocytes and have the contraction and expansion functions, it is recommended to use primary cells or induced pluripotent-derived cardiac cells to achieve a fully functional and beating cardiac construct for *in vivo* studies (Kaynak Bayrak and Gümüşderelioğlu, 2019).

## 5 Conclusion

Developing engineered cardiac tissues remains a challenge. A major challenge in CTE is to mimic the hierarchical structure of native cardiac tissue to create defined arrays of cells that show proper functional properties (Roberts et al., 2015). In this study, we have developed and optimized scaffolds with a novel design that simulates the orientation of fibres of the native human heart. It was found that these scaffolds show enhanced mechanical strength, lower degree of swelling, and higher percentage of cell survival compared to the lattice ones.

The significant contribution of this study is that Alg3Gel1 scaffolds mimicking the strand alignment angle of −70° to +70° could be an appropriate substrate for tissue engineering approaches where cardiac and/or endothelial cells are seeded on the scaffolds. Although the suitability of these scaffolds for extrusion-based bioprinting has not been explored, they might have the potential to be used for 3D-bioprinting, where the encapsulated hydrogels with cardiac cells as well as endothelial cells are 3D-printed together. This remains a subject for future studies. As a final remark, it is vital to mention that this study could be a primary foundation for future studies focusing on fabricating more complex cardiac constructs similar to cardiac tissue.

## Data Availability

The original contributions presented in the study are included in the article/Supplementary Material, further inquiries can be directed to the corresponding authors.
