# Supplementary material for: Optimization of 3D printing and in vitro characterization of alginate/gelatin lattice and angular scaffolds for potential cardiac tissue engineering
Source: Front Bioeng Biotechnol. 2023 May 25;11:1161804. doi: 10.3389/fbioe.2023.1161804 (PMC10248470; doi:10.3389/fbioe.2023.1161804)
Supplement: Supplementary file 1 [file Table1.DOCX]

Printability of scaffolds composed of Alg2Gel1 and Alg3Gel1 for both lattice and angular structures.

Statistical analysis performed via ANOVA-One way followed by Holm-Šídák's multiple comparisons test.

| **Holm-Šídák's multiple comparisons test** | Summary | Adjusted P Value |
| --- | --- | --- |
| Alg2Gel1[1.5,0.2]-L Vs Alg2Gel1[1.5,0.15]-L | * | 0.0295 |
| Alg2Gel1[1.5,0.2]-L Vs Alg2Gel1[1.5,0.1]-L | **** | <0.0001 |
| Alg2Gel1[1.5,0.2]-L Vs Alg2Gel1[1.3,0.2]-L | ns | 0.8047 |
| Alg2Gel1[1.5,0.2]-L Vs Alg2Gel1[1.3,0.15]-L | ns | 0.4185 |
| Alg2Gel1[1.5,0.2]-L Vs Alg2Gel1[1.3,0.1]-L | *** | 0.0001 |
| Alg2Gel1[1.3,0.15]-L Vs Alg2Gel1[1,0.2]-L | ns | 0.9761 |
| Alg2Gel1[1.3,0.1]-L Vs Alg2Gel1[1,0.15]-L | ns | 0.1494 |
| Alg2Gel1[1,0.2]-L Vs Alg2Gel1[1,0.1]-L | ns | 0.8234 |
| Alg2Gel1[1.5,0.15]-L Vs Alg2Gel1[1.5,0.1]-L | ns | 0.1863 |
| Alg2Gel1[1.5,0.15]-L Vs Alg2Gel1[1.3,0.2]-L | ns | 0.5040 |
| Alg2Gel1[1.5,0.15]-L Vs Alg2Gel1[1.3,0.15]-L | ns | 0.8608 |
| Alg2Gel1[1.5,0.15]-L Vs Alg2Gel1[1.3,0.1]-L | ns | 0.2225 |
| Alg2Gel1[1.5,0.15]-L Vs Alg2Gel1[1,0.2]-L | ns | 0.0664 |
| Alg2Gel1[1.5,0.15]-L Vs Alg2Gel1[1,0.15]-L | ns | 0.9761 |
| Alg2Gel1[1.5,0.15]-L Vs Alg2Gel1[1,0.1]-L | ns | 0.4753 |
| Alg2Gel1[1.5,0.1]-L Vs Alg2Gel1[1.3,0.2]-L | ** | 0.0025 |
| Alg2Gel1[1.5,0.1]-L Vs Alg2Gel1[1.3,0.15]-L | ** | 0.0099 |
| Alg2Gel1[1.5,0.1]-L vs Alg2Gel1[1.3,0.1]-L | ns | 0.9919 |
| Alg2Gel1[1.5,0.1]-L vs Alg2Gel1[1,0.2]-L | *** | 0.0002 |
| Alg2Gel1[1.5,0.1]-L vs Alg2Gel1[1,0.15]-L | * | 0.0379 |
| Alg2Gel1[1.5,0.1]-L Vs Alg2Gel1[1,0.1]-L | ** | 0.0022 |
| Alg2Gel1[1.3,0.2]-L Vs Alg2Gel1[1.3,0.15]-L | ns | 0.9761 |
| Alg2Gel1[1.3,0.2]-L Vs Alg2Gel1[1.3,0.1]-L | ** | 0.0030 |
| Alg2Gel1[1.3,0.2]-L Vs Alg2Gel1[1,0.2]-L | ns | 0.9012 |
| Alg2Gel1[1.3,0.2]-L vs Alg2Gel1[1,0.15]-L | ns | 0.8878 |
| Alg2Gel1[1.3,0.2]-L vs Alg2Gel1[1,0.1]-L | ns | 0.9919 |
| Alg2Gel1[1.3,0.15]-L Vs Alg2Gel1[1.3,0.1]-L | * | 0.0123 |
| Alg2Gel1[1.3,0.15]-L Vs Alg2Gel1[1,0.2]-L | ns | 0.6588 |
| Alg2Gel1[1.3,0.15]-L Vs Alg2Gel1[1,0.15]-L | ns | 0.9761 |
| Alg2Gel1[1.3,0.15]-L Vs Alg2Gel1[1,0.1]-L | ns | 0.9761 |
| Alg2Gel1[1.3,0.1]-L Vs Alg2Gel1[1,0.2]-L | *** | 0.0002 |
| Alg2Gel1[1.3,0.1]-L Vs Alg2Gel1[1,0.15]-L | * | 0.0468 |
| Alg2Gel1[1.3,0.1]-L Vs Alg2Gel1[1,0.1]-L | ** | 0.0026 |
| Alg2Gel1[1,0.2]-L Vs Alg2Gel1[1,0.15]-L | ns | 0.2969 |
| Alg2Gel1[1,0.2]-L Vs Alg2Gel1[1,0.1]-L | ns | 0.9031 |
| Alg2Gel1[1,0.15]-L Vs Alg2Gel1[1,0.1]-L | ns | 0.8794 |

| **Holm-Šídák's multiple comparisons test** | Summary | Adjusted P Value |
| --- | --- | --- |
| Alg2Gel1[1.5,0.2]-A Vs Alg2Gel1[1.5,0.15]-A | ns | 0.9709 |
| Alg2Gel1[1.5,0.2]-A Vs Alg2Gel1[1.5,0.1]-A | **** | <0.0001 |
| Alg2Gel1[1.5,0.2]-A Vs Alg2Gel1[1.3,0.2]-A | ns | 0.9752 |
| Alg2Gel1[1.5,0.2]-A Vs Alg2Gel1[1.3,0.15]-A | ns | 0.2124 |
| Alg2Gel1[1.5,0.2]-A Vs Alg2Gel1[1.3,0.1]-A | **** | <0.0001 |
| Alg2Gel1[1.3,0.15]-A Vs Alg2Gel1[1,0.2]-A | ns | 0.9752 |
| Alg2Gel1[1.3,0.1]-A Vs Alg2Gel1[1,0.15]-A | ns | 0.7680 |
| Alg2Gel1[1,0.2]-A Vs Alg2Gel1[1,0.1]-A | **** | <0.0001 |
| Alg2Gel1[1.5,0.15]-A Vs Alg2Gel1[1.5,0.1]-A | **** | <0.0001 |
| Alg2Gel1[1.5,0.15]-A Vs Alg2Gel1[1.3,0.2]-A | ns | 0.9752 |
| Alg2Gel1[1.5,0.15]-A Vs Alg2Gel1[1.3,0.15]-A | ns | 0.7419 |
| Alg2Gel1[1.5,0.15]-A Vs Alg2Gel1[1.3,0.1]-A | **** | <0.0001 |
| Alg2Gel1[1.5,0.15]-A Vs Alg2Gel1[1,0.2]-A | ns | 0.7778 |
| Alg2Gel1[1.5,0.15]-A Vs Alg2Gel1[1,0.15]-A | ns | 0.9752 |
| Alg2Gel1[1.5,0.15]-A Vs Alg2Gel1[1,0.1]-A | *** | 0.0001 |
| Alg2Gel1[1.5,0.1]-A Vs Alg2Gel1[1.3,0.2]-A | **** | <0.0001 |
| Alg2Gel1[1.5,0.1]-A Vs Alg2Gel1[1.3,0.15]-A | *** | 0.0002 |
| Alg2Gel1[1.5,0.1]-A vs Alg2Gel1[1.3,0.1]-A | ns | 0.9752 |
| Alg2Gel1[1.5,0.1]-A vs Alg2Gel1[1,0.2]-A | **** | <0.0001 |
| Alg2Gel1[1.5,0.1]-A vs Alg2Gel1[1,0.15]-A | **** | <0.0001 |
| Alg2Gel1[1.5,0.1]-A Vs Alg2Gel1[1,0.1]-A | ns | 0.8405 |
| Alg2Gel1[1.3,0.2]-A Vs Alg2Gel1[1.3,0.15]-A | ns | 0.2477 |
| Alg2Gel1[1.3,0.2]-A Vs Alg2Gel1[1.3,0.1]-A | **** | <0.0001 |
| Alg2Gel1[1.3,0.2]-A Vs Alg2Gel1[1,0.2]-A | ns | 0.9752 |
| Alg2Gel1[1.3,0.2]-A vs Alg2Gel1[1,0.15]-A | ns | 0.7820 |
| Alg2Gel1[1.3,0.2]-A vs Alg2Gel1[1,0.1]-A | **** | <0.0001 |
| Alg2Gel1[1.3,0.15]-A Vs Alg2Gel1[1.3,0.1]-A | ** | 0.0013 |
| Alg2Gel1[1.3,0.15]-A Vs Alg2Gel1[1,0.2]-A | ns | 0.0534 |
| Alg2Gel1[1.3,0.15]-A Vs Alg2Gel1[1,0.15]-A | ns | 0.9689 |
| Alg2Gel1[1.3,0.15]-A Vs Alg2Gel1[1,0.1]-A | ** | 0.0046 |
| Alg2Gel1[1.3,0.1]-A Vs Alg2Gel1[1,0.2]-A | **** | <0.0001 |
| Alg2Gel1[1.3,0.1]-A Vs Alg2Gel1[1,0.15]-A | *** | 0.0002 |
| Alg2Gel1[1.3,0.1]-A Vs Alg2Gel1[1,0.1]-A | ns | 0.9752 |
| Alg2Gel1[1,0.2]-A Vs Alg2Gel1[1,0.15]-A | ns | 0.3421 |
| Alg2Gel1[1,0.2]-A Vs Alg2Gel1[1,0.1]-A | **** | <0.0001 |
| Alg2Gel1[1,0.15]-A Vs Alg2Gel1[1,0.1]-A | *** | 0.0006 |

| **Holm-Šídák's multiple comparisons test** | Summary | Adjusted P Value |
| --- | --- | --- |
| Alg3Gel1[1.5,0.2]-L Vs Alg3Gel1[1.5,0.15]-L | *** | 0.0008 |
| Alg3Gel1[1.5,0.2]-L Vs Alg3Gel1[1.5,0.1]-L | **** | <0.0001 |
| Alg3Gel1[1.5,0.2]-L Vs Alg3Gel1[1.3,0.2]-L | ns | 0.9933 |
| Alg3Gel1[1.5,0.2]-L Vs Alg3Gel1[1.3,0.15]-L | *** | 0.0008 |
| Alg3Gel1[1.5,0.2]-L Vs Alg3Gel1[1.3,0.1]-L | **** | <0.0001 |
| Alg3Gel1[1.3,0.15]-L Vs Alg3Gel1[1,0.2]-L | ns | 0.9217 |
| Alg3Gel1[1.3,0.1]-L Vs Alg3Gel1[1,0.15]-L | ns | 0.1025 |
| Alg3Gel1[1,0.2]-L Vs Alg3Gel1[1,0.1]-L | **** | <0.0001 |
| Alg3Gel1[1.5,0.15]-L Vs Alg3Gel1[1.5,0.1]-L | **** | <0.0001 |
| Alg3Gel1[1.5,0.15]-L Vs Alg3Gel1[1.3,0.2]-L | ** | 0.0011 |
| Alg3Gel1[1.5,0.15]-L Vs Alg3Gel1[1.3,0.15]-L | ns | 0.9933 |
| Alg3Gel1[1.5,0.15]-L Vs Alg3Gel1[1.3,0.1]-L | **** | <0.0001 |
| Alg3Gel1[1.5,0.15]-L Vs Alg3Gel1[1,0.2]-L | ** | 0.0048 |
| Alg3Gel1[1.5,0.15]-L Vs Alg3Gel1[1,0.15]-L | ns | 0.2258 |
| Alg3Gel1[1.5,0.15]-L Vs Alg3Gel1[1,0.1]-L | **** | <0.0001 |
| Alg3Gel1[1.5,0.1]-L Vs Alg3Gel1[1.3,0.2]-L | **** | <0.0001 |
| Alg3Gel1[1.5,0.1]-L Vs Alg3Gel1[1.3,0.15]-L | **** | <0.0001 |
| Alg3Gel1[1.5,0.1]-L vs Alg3Gel1[1.3,0.1]-L | ns | 0.9933 |
| Alg3Gel1[1.5,0.1]-L vs Alg3Gel1[1,0.2]-L | **** | <0.0001 |
| Alg3Gel1[1.5,0.1]-L vs Alg3Gel1[1,0.15]-L | **** | <0.0001 |
| Alg3Gel1[1.5,0.1]-L Vs Alg3Gel1[1,0.1]-L | ns | 0.9370 |
| Alg3Gel1[1.3,0.2]-L Vs Alg3Gel1[1.3,0.15]-L | ** | 0.0011 |
| Alg3Gel1[1.3,0.2]-L Vs Alg3Gel1[1.3,0.1]-L | **** | <0.0001 |
| Alg3Gel1[1.3,0.2]-L Vs Alg3Gel1[1,0.2]-L | ns | 0.9370 |
| Alg3Gel1[1.3,0.2]-L vs Alg3Gel1[1,0.15]-L | ns | 0.1538 |
| Alg3Gel1[1.3,0.2]-L vs Alg3Gel1[1,0.1]-L | **** | <0.0001 |
| Alg3Gel1[1.3,0.15]-L Vs Alg3Gel1[1.3,0.1]-L | **** | <0.0001 |
| Alg3Gel1[1.3,0.15]-L Vs Alg3Gel1[1,0.2]-L | ** | 0.0048 |
| Alg3Gel1[1.3,0.15]-L Vs Alg3Gel1[1,0.15]-L | ns | 0.2258 |
| Alg3Gel1[1.3,0.15]-L Vs Alg3Gel1[1,0.1]-L | **** | <0.0001 |
| Alg3Gel1[1.3,0.1]-L Vs Alg3Gel1[1,0.2]-L | **** | <0.0001 |
| Alg3Gel1[1.3,0.1]-L Vs Alg3Gel1[1,0.15]-L | **** | <0.0001 |
| Alg3Gel1[1.3,0.1]-L Vs Alg3Gel1[1,0.1]-L | ns | 0.9207 |
| Alg3Gel1[1,0.2]-L Vs Alg3Gel1[1,0.15]-L | ns | 0.4186 |
| Alg3Gel1[1,0.2]-L Vs Alg3Gel1[1,0.1]-L | **** | <0.0001 |
| Alg3Gel1[1,0.15]-L Vs Alg3Gel1[1,0.1]-L | **** | <0.0001 |

| **Holm-Šídák's multiple comparisons test** | Summary | Adjusted P Value |
| --- | --- | --- |
| Alg3Gel1[1.5,0.2]-A Vs Alg3Gel1[1.5,0.15]-A | ** | 0.0018 |
| Alg3Gel1[1.5,0.2]-A Vs Alg3Gel1[1.5,0.1]-A | **** | <0.0001 |
| Alg3Gel1[1.5,0.2]-A Vs Alg3Gel1[1.3,0.2]-A | ns | 0.9959 |
| Alg3Gel1[1.5,0.2]-A Vs Alg3Gel1[1.3,0.15]-A | ns | 0.3468 |
| Alg3Gel1[1.5,0.2]-A Vs Alg3Gel1[1.3,0.1]-A | **** | <0.0001 |
| Alg3Gel1[1.3,0.15]-A Vs Alg3Gel1[1,0.2]-A | ns | 0.9959 |
| Alg3Gel1[1.3,0.1]-A Vs Alg3Gel1[1,0.15]-A | **** | <0.0001 |
| Alg3Gel1[1,0.2]-A Vs Alg3Gel1[1,0.1]-A | **** | <0.0001 |
| Alg3Gel1[1.5,0.15]-A Vs Alg3Gel1[1.5,0.1]-A | *** | 0.0003 |
| Alg3Gel1[1.5,0.15]-A Vs Alg3Gel1[1.3,0.2]-A | ** | 0.0018 |
| Alg3Gel1[1.5,0.15]-A Vs Alg3Gel1[1.3,0.15]-A | ns | 0.1000 |
| Alg3Gel1[1.5,0.15]-A Vs Alg3Gel1[1.3,0.1]-A | *** | 0.0003 |
| Alg3Gel1[1.5,0.15]-A Vs Alg3Gel1[1,0.2]-A | ** | 0.0031 |
| Alg3Gel1[1.5,0.15]-A Vs Alg3Gel1[1,0.15]-A | ns | 0.0838 |
| Alg3Gel1[1.5,0.15]-A Vs Alg3Gel1[1,0.1]-A | **** | <0.0001 |
| Alg3Gel1[1.5,0.1]-A Vs Alg3Gel1[1.3,0.2]-A | **** | <0.0001 |
| Alg3Gel1[1.5,0.1]-A Vs Alg3Gel1[1.3,0.15]-A | **** | <0.0001 |
| Alg3Gel1[1.5,0.1]-A vs Alg3Gel1[1.3,0.1]-A | ns | 0.9959 |
| Alg3Gel1[1.5,0.1]-A vs Alg3Gel1[1,0.2]-A | **** | <0.0001 |
| Alg3Gel1[1.5,0.1]-A vs Alg3Gel1[1,0.15]-A | ns | 0.1000 |
| Alg3Gel1[1.5,0.1]-A Vs Alg3Gel1[1,0.1]-A | **** | <0.0001 |
| Alg3Gel1[1.3,0.2]-A Vs Alg3Gel1[1.3,0.15]-A | ns | 0.3468 |
| Alg3Gel1[1.3,0.2]-A Vs Alg3Gel1[1.3,0.1]-A | **** | <0.0001 |
| Alg3Gel1[1.3,0.2]-A Vs Alg3Gel1[1,0.2]-A | ns | 0.9959 |
| Alg3Gel1[1.3,0.2]-A vs Alg3Gel1[1,0.15]-A | **** | <0.0001 |
| Alg3Gel1[1.3,0.2]-A vs Alg3Gel1[1,0.1]-A | **** | <0.0001 |
| Alg3Gel1[1.3,0.15]-A Vs Alg3Gel1[1.3,0.1]-A | **** | <0.0001 |
| Alg3Gel1[1.3,0.15]-A Vs Alg3Gel1[1,0.2]-A | ns | 0.4379 |
| Alg3Gel1[1.3,0.15]-A Vs Alg3Gel1[1,0.15]-A | *** | 0.0003 |
| Alg3Gel1[1.3,0.15]-A Vs Alg3Gel1[1,0.1]-A | **** | <0.0001 |
| Alg3Gel1[1.3,0.1]-A Vs Alg3Gel1[1,0.2]-A | **** | <0.0001 |
| Alg3Gel1[1.3,0.1]-A Vs Alg3Gel1[1,0.15]-A | ns | 0.1165 |
| Alg3Gel1[1.3,0.1]-A Vs Alg3Gel1[1,0.1]-A | **** | <0.0001 |
| Alg3Gel1[1,0.2]-A Vs Alg3Gel1[1,0.15]-A | **** | <0.0001 |
| Alg3Gel1[1,0.2]-A Vs Alg3Gel1[1,0.1]-A | **** | <0.0001 |
| Alg3Gel1[1,0.15]-A Vs Alg3Gel1[1,0.1]-A | **** | <0.0001 |
